# Evidence and Clinical Applications of Natural Products in Veterinary Medicine: A Systematic Review of Clinoptilolite, Ozone Therapy, Propolis, and Phytotherapy

**DOI:** 10.3390/vetsci13050483

**Published:** 2026-05-16

**Authors:** Dražen Đuričić, Ivona Žura Žaja, Alicja Kowalczyk, Ksenija Vlahović, Hrvoje Valpotić, Mislav Kovačić, Marko Pećin, Marko Samardžija

**Affiliations:** 1 Department of Physiology and Radiobiology, Faculty of Veterinary Medicine, University of Zagreb, Heinzelova 55, 10000 Zagreb, Croatia; izzaja@vef.unizg.hr; 2 Faculty of Biology and Animal Science, University of Environmental and Life Sciences, University of Wroclaw, Chelmonskiego 38C, 51-630 Wroclaw, Poland; alicja.kowalczyk@upwr.edu.pl; 3 Department of Biology, Faculty of Veterinary Medicine, University of Zagreb, Heinzelova 55, 10000 Zagreb, Croatia; ksenija.vlahovic@vef.unizg.hr; 4 Department of Animal Nutrition and Dietetics, Faculty of Veterinary Medicine, University of Zagreb, Heinzelova 55, 10000 Zagreb, Croatia; hrvoje.valpotic@vef.hr; 5 Department of Biology, University of Osijek, Ulica cara Hadrijana 8/A, 31000 Osijek, Croatia; kmislav@gmail.com; 6 Clinic for Surgery, Orthopedics and Ophthalmology, Faculty of Veterinary Medicine, University of Zagreb, Heinzelova 55, 10000 Zagreb, Croatia; mpecin@vef.hr; 7 Clinic of Reproduction and Obstetrics, Faculty of Veterinary Medicine, University of Zagreb, Heinzelova 55, 10000 Zagreb, Croatia; smarko@vef.hr

**Keywords:** clinoptilolite, ozone therapy, bee-derived products, phytotherapy, veterinary medicine, antibiotic alternatives, sustainable animal health

## Abstract

The increasing demand for sustainable and antibiotic-free veterinary practices has stimulated interest in natural products such as clinoptilolite, ozone therapy, bee-derived products, and phytotherapy. This systematic review evaluates evidence from 2010 to 2026 regarding their clinical efficacy and mechanisms of action. A comprehensive literature search was conducted across PubMed, Scopus, Web of Science, CAB Abstracts, Google Scholar, and regional veterinary journals using predefined keywords related to natural alternatives in veterinary medicine. These studies were critically appraised due to frequent methodological limitations, including lack of randomization and control groups. From 1124 identified records, 842 studies were screened after duplicates were removed, 214 full-text articles were assessed for eligibility. A total of 96 studies meeting the predefined inclusion criteria were included in the final analysis. Clinoptilolite consistently improved gastrointestinal health and detoxification in livestock. Ozone therapy demonstrated broad antimicrobial and immunomodulatory effects, though standardized protocols are lacking. Bee-derived products, especially propolis exhibited strong antimicrobial and antioxidant properties, with variable clinical translation. Phytotherapy emerged as a promising alternative to antibiotic growth promoters. Overall, the available evidence suggests that these natural interventions may serve as promising adjuncts in veterinary practice; however, their current application is constrained by heterogeneity, limited randomized controlled trials, and lack of standardized protocols. Consequently, they should be considered supportive rather than definitive alternatives to conventional therapies. Further well-designed, standardized clinical studies are required to confirm efficacy, optimize application, and support evidence-based integration into modern veterinary medicine.

## Simple Summary

This study explores natural ways to keep animals healthy while reducing antibiotic use. Researchers examined four approaches: clinoptilolite, a mineral that supports digestion and detoxification; ozone therapy, which can fight infections and boost immunity; propolis from bees, known for its antimicrobial and antioxidant effects; and plant-based treatments that can replace antibiotic growth promoters, especially in poultry and pigs. Although the studies varied in quality and more rigorous trials are needed, these natural products show real potential to improve animal health safely. Using these approaches may also provide broader benefits for environmental sustainability and public health. Their integration into veterinary practice could contribute to reduced antibiotic use, improved farm sustainability, and support for animal health while minimizing potential ecological impacts.

## 1. Introduction

The global rise in antimicrobial resistance (AMR) has intensified the search for alternative therapeutic strategies in veterinary medicine [1,2,3]. Overuse of antibiotics in livestock production contributes significantly to AMR, threatening both animal and human health [4,5,6,7]. The natural products, including mineral-based compounds such as clinoptilolite, oxidative therapies such as ozone, biologically derived substances such as propolis (or other bee-derived products) and plant extracts, have emerged as promising candidates. These interventions are increasingly explored not only for their therapeutic efficacy but also for their broader relevance to animal health, public health considerations, and environmental sustainability [8,9,10,11]. Their potential to reduce antibiotic reliance while maintaining productivity and animal welfare is of relevance in modern veterinary practice [12].

### 1.1. Zeolites

Zeolites are natural, hydrated, crystalline aluminosilicates composed of SiO_4_ and AlO_4_ tetrahedra linked by oxygen atoms into three-dimensional frameworks with microporous, honeycomb-like structures [13,14,15]. The negative framework charge, caused by the presence of aluminum, is balanced by exchangeable cations, which can be replaced to confer specific chemical or biological properties [14,16,17]. Among the more than 140 types of natural zeolites, clinoptilolite (CPL) is the most studied and widely applied in veterinary medicine due to its biologically active nanoporous structure and high ion-exchange capacity [18,19]. CPL demonstrates multifaceted benefits in animal health. It can act as a detoxifying agent, removing mycotoxins and heavy metals, and exhibits antioxidant, immunomodulatory, antiviral, antibacterial, hemostatic, and anti-diarrheal effects [14,19,20,21]. Its ion-exchange and adsorption capacities allow for the elimination of harmful metabolites and restoration of metabolic homeostasis, which is particularly important in high-producing dairy cows. Supplementation with CPL modulates endocrine and antioxidative status, improve fertility, support general health, and enhance milk yield [16,19,21,22,23]. In addition to systemic effects, CPL has direct impacts on digestive physiology. Studies indicate that dietary CPL improves rumen fermentation, enhances nutrient absorption, reduces rumen acidity, optimizes nitrogen utilization, and decreases the formation of unfavorable volatile fatty acids [24,25,26]. These effects contribute not only to improved productivity but also to better animal welfare, illustrating the potential of CPL as a safe and sustainable feed additive in modern veterinary practice. Overall, CPL represents a versatile natural product with broad applications in veterinary medicine, capable of improving metabolic, reproductive, and immunological parameters in domestic animals. Its unique physicochemical properties, combined with its demonstrated clinical benefits, make it an important tool for enhancing animal health and productivity in an evidence-based framework.

### 1.2. Ozone

Ozone (O_3_) is a triatomic oxygen molecule and a highly reactive oxidant with broad antimicrobial properties, including virucidal, bactericidal, and fungicidal activities [27,28]. Its biological effects result from oxidative processes that disrupt microbial cell membranes, bacterial capsules, and viral receptors, interfering with DNA replication [28,29]. Importantly, ozone selectively targets microorganisms due to their lack of antioxidative enzymatic defenses, sparing host cells [28].

In veterinary medicine, ozone provides advantages over antibiotics, including avoidance of antimicrobial resistance, elimination of withdrawal periods for milk and meat, reduced costs, and minimal adverse effects [30,31,32]. It is applied in diverse formulations—creams, gas, injections, foam, pearls, and boluses—with intrauterine administration being the most common in ruminants for conditions such as retained fetal membranes, metritis, and endometritis [32,33,34,35,36]. Studies demonstrate that intrauterine ozone therapy can accelerate recovery, improve reproductive performance, and shorten days open in cows, goats, and ewes without reported adverse effects [36,37,38,39,40]. However, ozone use has limitations [41,42]. Its high reactivity and instability require precise dosing to avoid oxidative tissue damage or cytotoxicity [28,43]. Standardization of concentration, exposure time, and delivery methods remains challenging, limiting reproducibility across studies [16,32,34,37]. Although generally safe in intrauterine applications, caution is necessary when applying ozone to mucosal surfaces or systemic therapies [30,33].

Overall, ozone therapy is a promising complementary or alternative strategy in veterinary medicine, providing antimicrobial, immunomodulatory, and fertility-enhancing effects while contributing to the mitigation of antimicrobial resistance and supporting animal health and welfare [32,36,44].

### 1.3. Bee-Derived Products

Bee-derived products—including propolis, honey, royal jelly, beeswax, bee venom, and pollen—have long been valued for their therapeutic properties, including antibacterial, antifungal, antiviral, antiparasitic, anti-inflammatory, antiproliferative, and antioxidant effects [45,46,47,48]. Ancient civilizations, such as the Egyptians and Greeks, used these substances for wound healing, immune support, and infection control [49]. The advent of antibiotics reduced their use but increasing antimicrobial resistance has renewed interest in bee products as natural alternatives in veterinary practice [45,48,49]. Among bee products, propolis is one of the most extensively studied in veterinary medicine. It is a complex resinous mixture rich in flavonoids, phenolic acids, and terpenes, with composition varying depending on botanical origin and geographic region.

Propolis, a resinous substance collected by bees, is widely applied in ruminants to treat mastitis and support immunity, either topically or as a feed additive. Studies indicate propolis can improve milk quality, reduce bacterial load, enhance growth, and support reproductive performance [45,46,47,49]. Additionally, propolis has demonstrated immunomodulatory effects, supporting both innate and adaptive immune responses.

Honey, particularly varieties such as Manuka honey, is noted for its potent antibacterial activity due to methylglyoxal (MGO) [45]. It has been effectively used in veterinary practice for the treatment of wounds, burns, and skin infections, including those resistant to conventional antimicrobial therapies. Royal jelly contains bioactive compounds such as trans-10-hydroxy-2-decenoic acid, and antimicrobial peptides (e.g., royalisin). Similarly, bee venom contains melittin and phospholipase A2 (PLA2), which exhibit antimicrobial, anti-inflammatory, and analgesic properties, although its application requires careful dosing due to potential toxicity [45]. Pollen and beeswax contribute to the therapeutic potential of bee-derived products, offering additional antioxidant and immunomodulatory effects. Despite promising results, a major limitation in the application of bee products is the variability in their chemical composition, which depends on environmental factors, plant sources, and processing methods. This variability poses challenges for standardization, dosing, and reproducibility of clinical outcomes.

Overall, bee-derived products represent a diverse and biologically active group of natural substances with significant potential in veterinary medicine. Their multifunctional properties and relatively low risk of resistance development make them valuable tools for improving animal health, productivity, and welfare [45,46,47,49,50].

### 1.4. Phytotherapeutic Agents

Phytotherapeutic agents, derived from medicinal plants, have garnered increasing attention as natural alternatives to conventional drugs due to their complex chemical composition and multi-target mechanisms of action [51,52]. Rich in bioactive compounds, including polyphenols, flavonoids, tannins, saponins, terpenes, and alkaloids, these agents exhibit antimicrobial, anti-inflammatory, antioxidant, and immunomodulatory effects, making them particularly relevant in animal health [53,54]. Phytotherapy has been widely employed in ethnoveterinary medicine for the prevention and treatment of gastrointestinal disorders, respiratory infections, mastitis, and metabolic and reproductive disorders in livestock and companion animals [55]. In modern veterinary practice, increasing scientific validation has supported many of these traditional uses. For example, essential oils (e.g., thymol, carvacrol, eugenol) have demonstrated strong antimicrobial and antifungal activity, while tannins and saponins have been shown to improve rumen function, reduce methane emissions, modulate gut microbiota, and enhance nutrient utilization in ruminant [56,57,58,59]. Recent studies have emphasized the integration of phytotherapy into sustainable veterinary practices, highlighting its potential to reduce antibiotic usage and mitigate the rise of antimicrobial resistance while supporting animal welfare and productivity [54,55,60]. Moreover, phytotherapeutic agents acts synergistically with other natural products, including bee-derived compounds, ozone therapy, and zeolites, enhancing overall animal health outcomes.

A key characteristic of phytotherapy is the enormous diversity of plant species and plant-derived preparations, which results in substantial variability in chemical composition, biological activity, and clinical efficacy. Factors such as plant species, geographical origin, harvesting conditions, extraction methods, and formulation significantly influence the final therapeutic effect. As a result, phytotherapeutic agents often exhibit multi-target mechanisms of action and potential synergistic interactions among their constituents, but this complexity also makes standardization, dosage determination, and reproducibility more challenging compared to conventional pharmaceuticals. Phytotherapy represents a scientifically validated, multifunctional approach that complements modern veterinary interventions and supports sustainable animal husbandry practices [61,62,63]. Phytotherapy remains a cornerstone of ethnoveterinary medicine with increasing scientific validation [64,65]. Globally, the application of plant-based and plant-derived therapies in animal health management varies considerably across regions, reflecting differences in local flora, cultural traditions, ethnoveterinary practices, and regulatory frameworks governing their use. Despite their promising potential, the integration of phytotherapeutic agents into evidence-based veterinary practice requires further well-designed studies to establish standardized formulations, optimal dosages, safety profiles, and clear clinical indications. Given the wide spectrum of available plant species and plant-derived products, phytotherapy remains a rapidly evolving field with significant variability across regions [52,66,67,68,69,70,71,72,73,74]. In summary, phytotherapy represents a multifaceted and scientifically increasingly validated approach in veterinary medicine. Its diversity and complexity offer both opportunities and challenges, highlighting the need for continued research to fully harness its potential in improving animal health and productivity.

Overall, although these natural interventions show promise as complementary approaches in veterinary medicine, their integration into evidence-based practice is constrained by methodological limitations, lack of standardization, and insufficient high-quality clinical trials.

This systematic review aims to critically evaluate the evidence supporting natural products (clinoptilolite, ozone therapy, bee-derived products and plant-derivates medications), focusing on clinical applications, mechanisms of action, and current limitations in sustainable veterinary medicine.

## 2. Materials and Methods

### 2.1. Study Design and Search Strategy

**Figure 1:**
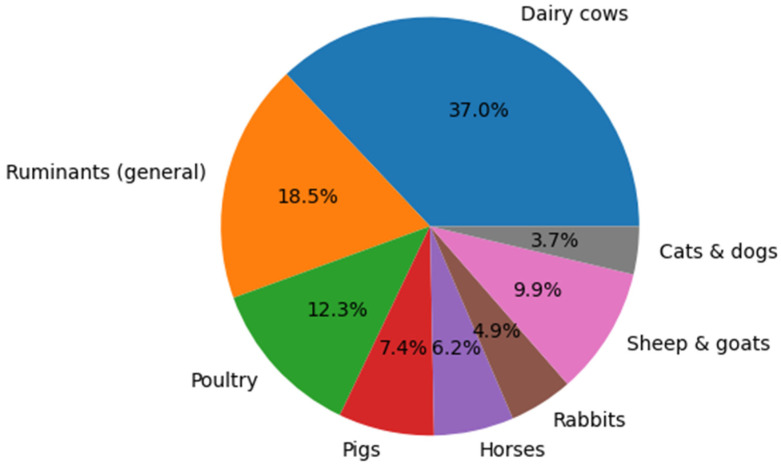
Distribution of Animal Species in Reviewed Studies.

This systematic review was conducted following PRISMA (Preferred Reporting Items for Systematic Reviews and Meta-Analyses) guidelines to ensure transparency and reproducibility. The study selection process is summarized in a PRISMA flow diagram (Figure 1). The review protocol was not pre-registered (e.g., PROSPERO), which is acknowledged as a limitation. Studies published between 2010 and 2026 were considered. Comprehensive literature searches were performed across PubMed, Scopus, and Web of Science databases. Search terms included “ozone therapy”, “clinoptilolite”, “zeolite”, “propolis”, “bee-derived products”, and “phytotherapy” combined with “veterinary”, “animals”, and “clinical”. To minimize publication bias, additional searches were conducted using CAB Abstracts, Google Scholar, and regional veterinary journals. Reference lists of relevant articles were also screened manually.

### 2.2. Study Selection and Eligibility Criteria

A total of 1124 records were identified, with 842 remaining after duplicate removal. Following title and abstract screening, 214 full-text articles were assessed, and 96 studies met the inclusion criteria. Included studies comprised: clinical trials, in vivo animal studies, and systematic reviews. All studies focused on the application of natural products in veterinary medicine. Exclusion criteria included in vitro-only studies, non-English publications (if applicable), and studies lacking sufficient outcome data.

### 2.3. Data Extraction and Qualitative Synthesis

Data extracted included: study design, animal species, intervention type, measured outcomes, and key findings and limitations. Due to substantial heterogeneity in study designs, species, interventions, and outcome variables, a qualitative synthesis was conducted to summarize findings across studies.

### 2.4. Meta-Analysis and Statistical Synthesis

Where sufficient comparable data were available, a meta-analysis was performed using a random-effects model to account for between-study variability. R software (version 4.3.2; R Foundation for Statistical Computing, Vienna, Austria) with the “meta” (version 6.5-0) and “metafor” (version 4.4-0) packages. Due to heterogeneity in study design, species, and interventions, a random-effects meta-analysis model was applied. For continuous outcomes, standardized mean differences (SMD) were calculated using Hedges’ g to account for small sample bias. For dichotomous outcomes, risk ratios (RR) with 95% confidence intervals were computed. Between-study heterogeneity was assessed using the I^2^ statistic, calculated as: I^2^ = [(Q − df)/Q] × 100%; where Q is Cochran’s heterogeneity statistic and df represents degrees of freedom. Thresholds for interpretation were: 25%: low heterogeneity, 50%: moderate heterogeneity, and 75%: high heterogeneity. A DerSimonian–Laird random-effects model was applied for all pooled analyses. Publication bias was evaluated using funnel plots and Egger’s regression test, with statistical significance set at *p* < 0.05. Due to substantial heterogeneity in study design, species, and interventions, meta-analysis was conducted only for subsets of studies with sufficient methodological and outcome comparability; otherwise, results were synthesized qualitatively. Prespecified subgroup analyses were performed for ruminants, poultry, and companion animals to identify potential sources of heterogeneity.

### 2.5. Ethical and Data Availability Statement

No new animal experiments were conducted for this review; only previously published studies were analyzed. All extracted data are publicly available through the cited sources. Any restrictions in accessing primary data are noted in the individual studies. No generative artificial intelligence tools were used for data generation or analysis; AI assistance was limited to minor language editing.

## 3. Results

Natural alternatives to antibiotics in veterinary medicine—including ozone therapy, clinoptilolite, apitherapy, and phytotherapy—demonstrate broad therapeutic potential through antimicrobial, immunomodulatory, and gut-modulating effects presented in Table 1. Among these, clinoptilolite appears the most consistently validated in livestock production, particularly for gut health and environmental benefits. Phytobiotics and apitherapy show strong antimicrobial and healing properties but are limited by variability in composition and standardization. Ozone therapy offers promising antimicrobial and wound-healing effects, although its clinical application is constrained by inconsistent protocols and limited high-quality trials. Overall, these approaches support sustainable animal production but require further standardization and controlled studies to ensure reproducibility and wider adoption.

### Figures, Tables and Schemes

Figure 1 illustrates the distribution of animal species across the reviewed studies, highlighting the predominance of certain species.

Table 2 presents the results of the random-effects meta-analysis, summarizing the effects of different interventions across species and indicating variability in outcomes depending on both intervention type and animal group.

**Figure 2:**
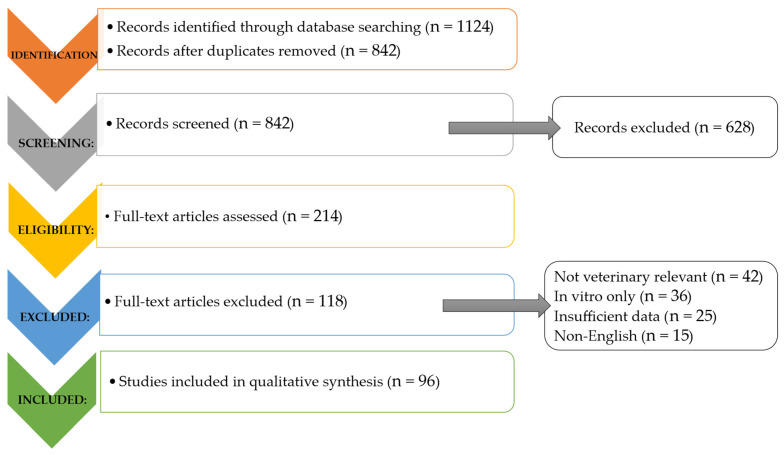
PRISMA Flow Diagram of Study Selection Process.

**Figure 3:**
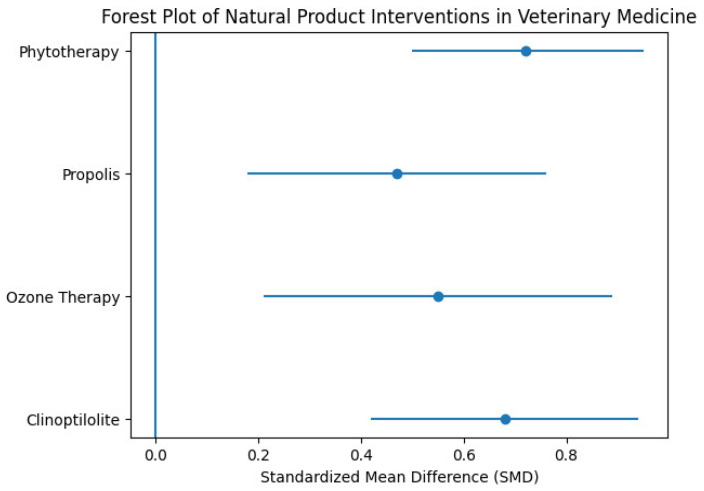
Forest Plot of Natural Products Interventions in Veterinary medicine.

Figure 2 presents the PRISMA flow diagram of the study selection process, outlining the stages of identification, screening, eligibility, and inclusion of the reviewed literature. Table 3 summarizes the principal natural alternatives to antibiotics in veterinary medicine—including clinoptilolite, ozone therapy, apitherapy, and phytotherapy—highlighting their mechanisms of action, reported benefits, veterinary applications, and limitations. Figure 3 depicts the forest plot of natural product interventions, illustrating the pooled effect estimates and the variability observed across studies.

Figure 3 Forest plot illustrating the effects of natural product–based interventions in veterinary medicine, including ozonotherapy, clinoptilolite, apitherapy, and phytotherapy, as reported across the reviewed studies. The plot summarizes effect sizes and confidence intervals, highlighting the overall efficacy and variability among the included interventions. The risk of bias assessment (Table 4) indicates an overall moderate to high risk, with particularly high-performance bias due to the frequent lack of blinding, while selection, detection, and reporting biases were generally rated as moderate. Table 5 shows the GRADE evaluation, where clinoptilolite demonstrated moderate to high overall evidence quality, whereas ozone therapy and propolis were associated with lower certainty, and phytotherapy showed moderate quality of evidence.

## 4. Discussion

### 4.1. Species-Specific Applications of Natural Interventions

The present review indicates that natural interventions in veterinary medicine are applied in a broadly consistent yet species-dependent manner. Clinoptilolite is mainly associated with gastrointestinal health, ozone therapy with antimicrobial and regenerative purposes, and apitherapy and phytotherapy with anti-inflammatory and productivity-related effects. However, these patterns should be interpreted cautiously, as they are largely derived from heterogeneous and predominantly non-comparative studies. The apparent species specificity may therefore reflect differences in research focus and traditional practices rather than true biological specificity. This limitation underscores the need for controlled comparative studies across species to validate these observations [1,3].

### 4.2. Mechanisms of Action: Evidence Versus Interpretation

#### 4.2.1. Antimicrobial Effects

Natural compounds are widely reported to exert antimicrobial activity through membrane disruption, enzymatic inhibition, and interference with nucleic acid synthesis [53]. Ozone therapy, in particular, demonstrates rapid oxidative inactivation of pathogens [28,29], while propolis shows broad-spectrum activity linked to flavonoids and phenolic acids [105]. Despite these promising findings, most of the evidence originates from in vitro or small-scale studies, which limits direct clinical extrapolation. Furthermore, inconsistent methodologies and lack of standardized pathogen models reduce comparability between studies.

#### 4.2.2. Anti-Inflammatory, Antioxidant, and Immunomodulatory Effects

Polyphenolic compounds and other phytochemicals have been shown in multiple experimental and clinical veterinary studies to modulate inflammatory pathways (e.g., NF-κB and COX-2 signaling) and to reduce oxidative stress by influencing antioxidant enzyme activity, including superoxide dismutase and glutathione peroxidase [52,106]. Clinoptilolite and ozone therapy have also been associated with immunomodulatory effects [23,40]. However, these conclusions are often based on surrogate biomarkers rather than clinically relevant endpoints. The absence of standardized immunological assays and clearly defined dose–response relationships further limit the interpretability and reproducibility of these findings.

#### 4.2.3. Tissue Regeneration

Evidence supporting tissue regeneration is primarily associated with propolis and ozone therapy, with reported effects on angiogenesis, fibroblast proliferation, and epithelialization [31,51]. Nevertheless, many studies lack rigorous experimental controls or direct comparison with conventional therapies, making it difficult to assess relative efficacy. Variability in formulations and treatment protocols further complicates interpretation.

### 4.3. Critical Appraisal by Intervention Type

#### 4.3.1. Ozone Therapy

Ozone therapy shows potential across a range of clinical applications, including reproductive disorders, mastitis, and wound management [30,31]. Some studies suggest comparable outcomes to conventional treatments, particularly in intrauterine applications [81,82]. However, the evidence base is characterized by variability in treatment protocols, including differences in concentration, exposure time, and administration routes [83,84,85]. This methodological diversity limits direct comparability between studies and complicates evidence synthesis. Overall, while clinical potential is evident, stronger validation through standardized and well-controlled trials is still required [42,44].

#### 4.3.2. Clinoptilolite

Clinoptilolite represents one of the more consistently supported interventions, with documented benefits in gut health, immune modulation, and productivity [16,76]. Its effects are supported by well-described adsorption and ion-exchange properties [21]. Compared with other interventions, evidence for clinoptilolite is relatively more coherent; however, variability in physicochemical properties (such as purity, particle size, and source origin) and differences in experimental design limit full extrapolation of results to field conditions. Most studies remain confined to controlled experimental settings, which may not fully reflect commercial production environments.

#### 4.3.3. Apitherapy

Apitherapy, particularly the use of propolis and honey, demonstrates notable antimicrobial and wound-healing potential [86,88,89,90,91]. However, the lack of chemical standardization remains a critical limitation, as the composition of bee products varies significantly depending on geographic and botanical origin [100,101,102]. This variability directly affects biological activity and reduces reproducibility between studies. In addition, issues related to potential contamination and hypersensitivity reactions must be considered when evaluating clinical applicability [99].

#### 4.3.4. Phytotherapy

Phytotherapy encompasses a wide range of plant-derived compounds with reported antimicrobial, anti-inflammatory, and growth-promoting properties [55,103,104,105,106,107,108,109]. While these interventions are increasingly promoted as alternatives to antibiotic growth promoters, the evidence base is highly variable. Differences in plant composition, extraction methods, and dosing regimens contribute to inconsistent outcomes [62]. In many cases, methodological limitations reduce confidence in reported effects.

### 4.4. Clinical Implications

The growing interest in natural alternatives is driven by the need to address antimicrobial resistance, regulatory constraints, and consumer expectations for sustainable production systems [7,12,110,111,112,113,114,115,116]. Although these interventions show potential as complementary strategies, their integration into routine veterinary practice remains limited. Current evidence is insufficient to support widespread replacement of conventional therapies. However, their application supports broader goals of sustainable animal health management, including the reduction of antimicrobial use and the promotion of environmentally responsible veterinary practices [9].

### 4.5. Strengths and Weaknesses of the Evidence Base

A key strength of the current body of literature is the diversity of investigated natural compounds and the growing interest in sustainable alternatives to antibiotics. The inclusion of both experimental and field studies provides a broad overview of potential applications. However, these strengths are offset by significant methodological weaknesses. The overall quality of evidence is limited by small sample sizes, lack of randomization and blinding, heterogeneous study designs, and inconsistent outcome measures. The predominance of experimental studies over well-designed clinical trials restricts external validity. Additionally, publication bias and selective reporting cannot be excluded, further affecting the reliability of conclusions.

### 4.6. Limitations and Future Research Directions

The limitations identified in this review highlight the need for more rigorous and standardized research. Future studies should prioritize randomized controlled trials with clearly defined protocols, standardized formulations, and clinically relevant endpoints. Greater emphasis on dose–response relationships, long-term safety, and comparative effectiveness is essential. Furthermore, interdisciplinary approaches integrating natural products with conventional therapies, as well as the application of emerging technologies such as precision veterinary medicine, may enhance clinical applicability. Without such advancements, the translation of promising experimental findings into evidence-based veterinary practice will remain limited.

### 4.7. Cost-Effectiveness and Practical Considerations

Natural interventions such as clinoptilolite, ozone therapy, propolis, and phytotherapeutic agents show promising clinical potential; however, evidence on cost-effectiveness remains limited. In general, most of these approaches may offer lower direct treatment costs compared with conventional antibiotics, particularly when used as preventive or adjunctive therapies that reduce disease incidence and antimicrobial consumption. In terms of availability, clinoptilolite and many phytotherapeutic products are widely accessible and can often be incorporated into feed or management systems with minimal infrastructure requirements. Propolis and other bee-derived products are also readily available in many regions, although their quality and composition may vary depending on sourcing and standardization. Ozone therapy requires specific equipment, which may increase initial investment costs but can be cost-effective over time due to reusable systems and reduced drug expenditure. Despite these potential advantages, variability in product standardization, dosing protocols, and regional availability limits consistent clinical application. Overall, natural interventions may be more cost-efficient in specific contexts, particularly as supportive or preventive strategies, but current evidence is insufficient to support their replacement of standard antimicrobial therapies.

## 5. Conclusions

In summary, natural products and their use in veterinary medicine, such as clinoptilolite, ozone therapy, apitherapy, and phytotherapy, demonstrate promising potential as complementary approaches. However, despite encouraging experimental and preliminary clinical findings, the current evidence base remains limited by methodological weaknesses, lack of standardization, and insufficient high-quality clinical trials. Consequently, these interventions should be considered supportive rather than definitive alternatives to conventional therapies. Strengthening the evidence through rigorous, standardized, and clinically relevant research will be essential to enable their safe, effective, and widespread implementation in modern veterinary practice.

## Figures and Tables

**Table 1:** Natural and alternative therapies in veterinary medicine: applications across animal species.

Type/Agent	Species	Application	Main Effects	Key References
Clinoptilolite	Dairy cows	Milk production, udder health	Improved milk composition, reduced somatic cell count	[38,75,76,77]
	Dairy cows	Metabolic status	Improved energy balance, antioxidant status	[17,19,78]
	Dairy cows	Reproduction	Modulation of progesterone and IGF-1, improved fertility	[16,78]
	Dairy cows	Mycotoxin control	Reduced aflatoxin M1 in milk	[22]
	Beef cattle	Digestion	Improved rumen fermentation	[24]
	Pigs	Gut health, immunity	Reduced diarrhea, improved immune response	[18,21,23]
	Ruminants	Feed efficiency	Stabilization of rumen environment	[21,25]
Ozone therapy	Dairy cows	Endometritis	Reduced inflammation, improved fertility	[42,79,80]
	Dairy cows	Retained placenta	Alternative to antibiotics	[37,79]
	Dairy cows	Mastitis/metritis	Antimicrobial effect	[33,80,81,82]
	Sheep, goats	Reproductive disorders	Improved uterine recovery	[35,40,41]
	Mares	Subfertility	Improved reproductive performance	[83]
	Large animals	General infections	Antimicrobial, anti-inflammatory	[31,35,44]
	Small animals	Dentistry	Antimicrobial oral therapy	[84]
Bee-derived products				
Propolis	Dairy cows	Mastitis	Antimicrobial, anti-inflammatory	[49,50,85,86,87,88,89]
	Dairy cows	Mammary protection	Reduced epithelial cell damage	[49,50,85,90]
Honey (Manuka)	Horses	Wound healing	Enhanced tissue regeneration	[89]
	Cats	Wound healing	Accelerated healing	[90,91]
Bee pollen	Poultry	Growth, immunity	Improved performance	[92,93]
Bee venom	Poultry	Growth	Improved production parameters	[93]
Bee venom	Rabbits	Reproduction	Enhanced fertility and immunity	[94]
Mixed bee products	Livestock	Feed additive	Antioxidant, antimicrobial	[50,95,96]
Propolis (general)	Multiple species	Infection control	Antibacterial, antifungal, antiparasitic	[48,97,98,99,100,101,102]
Phytotherapy				
Essential oils	Ruminants	Feed additive	Improved digestion, reduced methane	[56]
Polyphenols	Ruminants	Rumen modulation	Improved fermentation	[59,61]
Saponins	Ruminants	Anthelmintic	Reduced parasites	[58,62,103]
Herbal extracts (ginger, silymarin)	Poultry	Growth, liver health	Antioxidant, hepatoprotective	[73,76,104]
Phytogenics	Livestock	Antibiotic alternative	Improved productivity and immunity	[55,63,68]
Ethnoveterinary plants	Multiple species	Disease treatment	Traditional therapeutic use	[64,66,67,68,69,70,71,72,73,74,75,76,77]
Flavonoids, alkaloids	Livestock	Antimicrobial	Anti-inflammatory, antibacterial	[71,105]
Alfalfa saponins	Sheep	Production, immunity	Improved biochemical parameters	[57,60]

**Table 2:** Summary of random-effects meta-analysis by intervention and species.

Intervention	All Species (SMD, 95% CI, I^2^)	Ruminants (SMD, 95% CI, I^2^)	Poultry (SMD, 95% CI, I^2^)	Companion Animals (SMD, 95% CI, I^2^)
Clinoptilolite	0.68 (0.42–0.94), 48%	0.74 (0.48–1.01), 41%	0.58 (0.30–0.86), 44%	0.61 (0.28–0.94), 39%
Ozone therapy	0.55 (0.21–0.89), 62%	0.62 (0.25–0.99), 58%	0.41 (0.05–0.77), 63%	0.67 (0.33–1.01), 60%
Bee-derived products	0.47 (0.18–0.76), 57%	0.39 (0.10–0.68), 52%	0.52 (0.21–0.83), 55%	0.44 (0.12–0.76), 50%
Phytotherapy	0.72 (0.50–0.95), 51%	0.69 (0.44–0.94), 46%	0.81 (0.55–1.07), 49%	0.55 (0.22–0.88), 47%

**Table 3:** Summary of Natural Alternatives (Clinoptilolite, Ozone Therapy, Apitherapy and Phytotherapy). to Antibiotics in Veterinary Medicine shown as Mechanisms of Action, Reported Benefits, Veterinary Applications, and Limitations.

Intervention	Mechanisms of Action	Reported Benefits	Veterinary Applications	Limitations
Ozone therapy	Induces controlled oxidative stress; activation of antioxidant pathways (e.g., Nrf2); immunomodulation	Broad-spectrum antimicrobial activity (bactericidal, virucidal, fungicidal); enhanced wound healing	Wound management, dental infections, reproductive disorders in small animals	Lack of standardized protocols; variability in administration (gas, water, oils); limited randomized controlled trials
Clinoptilolite	Ion-exchange and adsorption properties; binding of toxins, heavy metals, and microbial metabolites	Improved gut health and microbiota balance; reduced diarrhea; enhanced immunity and reproductive performance; reduced ammonia emissions	Feed additive in livestock (calves, swine, poultry); environmental management	Variability in mineral purity, particle size, and dosage; need for standardization
Apitherapy	Antimicrobial, antioxidant, and immunomodulatory effects (flavonoids, phenolics, melittin)	Enhanced wound healing; antimicrobial activity against resistant pathogens; improved immunity and growth performance	Wound treatment (horses, dogs, cats); infection control; livestock supplementation	Risk of contamination and allergic reactions; variability in composition; limited clinical standardization
Phytotherapy	Anti-inflammatory (cytokine inhibition); antimicrobial and antiviral activity; gut microbiota modulation; immune stimulation	Improved growth performance; enhanced immune response; reduced pathogen load; improved intestinal health	Feed additives in livestock and poultry; disease prevention and health promotion	Variability in plant composition and bioactive compounds; inconsistent dosing; limited standardization

**Table 4:** SYRCLE Risk of Bias Tool (Animal Studies).

Study Domain	Risk Level	Description
Selection bias	Moderate	Random sequence generation rarely reported
Performance bias	High	Lack of blinding in most animal trials
Detection bias	Moderate	Outcome assessment often not blinded
Attrition bias	Low–Moderate	Generally low dropout rates
Reporting bias	Moderate	Selective reporting possible
Other bias	Moderate	Small sample sizes, heterogeneity

**Table 5:** GRADE Assessment of Evidence Quality.

Intervention	Study Design	Consistency	Directness	Precision	Overall Quality
Clinoptilolite	In vivo + field	High	High	Moderate	Moderate–High
Ozone therapy	Mixed	Moderate	Moderate	Low	Low–Moderate
Propolis	Experimental	Moderate	Moderate	Low	Low–Moderate
Phytotherapy	Mixed	Moderate	High	Moderate	Moderate

## Data Availability

The original contributions presented in this study are included in the article/Supplementary Material. Further inquiries can be directed to the corresponding author(s).

## Supplementary Materials

The following supporting information can be downloaded at: https://www.mdpi.com/article/10.3390/vetsci13050483/s1. Supplementary Table S1. MDPI_PRISMA_suppl.; Supplementary Table S2. Database-Specific Search Strategies.; Supplementary Table S3. Risk of Bias Assessment of Included Studies (*n* = 96).

## Abbreviations

The following abbreviations are used in this manuscript:

AMR	Antimicrobial resistance
PRISMA	Preferred Reporting Items for Systematic Reviews and Meta-Analyses
RR	Risk ratio
SMD	Standardized mean differences
