# Supplementary material for: Evidence and Clinical Applications of Natural Products in Veterinary Medicine: A Systematic Review of Clinoptilolite, Ozone Therapy, Propolis, and Phytotherapy
Source: Vet Sci. 2026 May 16;13(5):483. doi: 10.3390/vetsci13050483 (PMC13211563; doi:10.3390/vetsci13050483)
Supplement: Supplementary file 1 [file vetsci-13-00483-s001.zip › Table S1 MDPI_PRISMA_suppl_S1.pdf]

Reference	First Autho	Publicatio	Study Inclu	PRISMA Eligibility
1	Rizzo	2023	Yes	Eligible
2	Wang	2024	Yes	Eligible
3	Salman	2026	Yes	Eligible
4	Marshall	2011	Yes	Eligible
5	Landers	2012	Yes	Eligible
6	Ventola	2015	Yes	Eligible
7	Matheou	2025	Yes	Eligible
8	Mackenzie	2019	Yes	Eligible
9	Prestinaci	2020	Yes	Eligible
10	Ekor	2014	Yes	Eligible
11	Laurino	2015	Yes	Eligible
12	Oggiano	2023	Yes	Eligible
13	Alotaibi	2025	Yes	Eligible
14	Đuričić	2020	Yes	Eligible
15	Folnožić	2019	Yes	Eligible
16	Vince	2022	Yes	Eligible
17	Maity	2021	Yes	Eligible
18	Valpotić	2017	Yes	Eligible
19	Katsoulos	2016	Yes	Eligible
20	Travagli	2010	Yes	Eligible
21	Bocci	2020	Yes	Eligible
22	Abd El-Aziz	2023	Yes	Eligible
23	Banerjee	2024	Yes	Eligible
24	Sciorsci	2020	Yes	Eligible
25	Abdelnour	2019	Yes	Eligible
26	de Souza	2021	Yes	Eligible
27	Abo-El-Soc	2018	Yes	Eligible
28	Đuričić	2015	Yes	Eligible
29	Đuričić	2012	Yes	Eligible
30	Đuričić	2018	Yes	Eligible
31	Zobel	2012	Yes	Eligible
32	Đuričić	2016	Yes	Eligible
33	Đuričić	2015	Yes	Eligible
34	Đuričić	2011	Yes	Eligible
35	Đuričić	2020	Yes	Eligible
36	Elvis	2011	Yes	Eligible
37	Orlandin	2021	Yes	Eligible
38	Di Paolo	2004	Yes	Eligible
39	Rubin	2025	Yes	Eligible
40	Abu-Seida	2023	Yes	Eligible
41	Bačić	2016	Yes	Eligible
42	Šuran	2020	Yes	Eligible
43	Özdemir	2022	Yes	Eligible

44	Abd El-Aziz	2023	Yes	Eligible
45	Kuralkar	2021	Yes	Eligible
46	Yang	2015	Yes	Eligible
47	Pérez-Flore	2025	Yes	Eligible
48	Alagawany	2021	Yes	Eligible
49	Yang	2022	Yes	Eligible
50	Kholif	2023	Yes	Eligible
51	Priyashant	2026	Yes	Eligible
52	Quintavalla	2024	Yes	Eligible
53	Tamminen	2018	Yes	Eligible
54	Alasbahi	2022	Yes	Eligible
55	Oliveira	2021	Yes	Eligible
56	Mayer	2014	Yes	Eligible
57	Aziz	2020	Yes	Eligible
58	Dong	2021	Yes	Eligible
59	Schlittenla	2022	Yes	Eligible
60	Holzner	2024	Yes	Eligible
61	Wendimu	2024	Yes	Eligible
62	Đurić Jarić	2025	Yes	Eligible
63	Folnožić	2019	Yes	Eligible
64	Samardžija	2017	Yes	Eligible
65	Samardžija	2021	Yes	Eligible
66	Samardžija	2025	Yes	Eligible
67	Carbonari	2026	Yes	Eligible
68	Escandón	2020	Yes	Eligible
69	Tomanić	2023	Yes	Eligible
70	Moroni	2026	Yes	Eligible
71	Melanie	2024	Yes	Eligible
72	Chuttong	2023	Yes	Eligible
73	da Silva	2026	Yes	Eligible
74	Mikniene	2026	Yes	Eligible
75	Wang	2016	Yes	Eligible
76	Bischofber	2016	Yes	Eligible
77	Abdelnour	2019	Yes	Eligible
78	Han	2010	Yes	Eligible
79	Elkomy	2021	Yes	Eligible
80	Aguiar	2014	Yes	Eligible
81	Santos	2020	Yes	Eligible
82	Stevanović	2024	Yes	Eligible
83	Bankova	2021	Yes	Eligible
84	Zulhendri	2021	Yes	Eligible
85	Maestrini	2020	Yes	Eligible
86	Saeed	2017	Yes	Eligible
87	Gutiérrez-(	2020	Yes	Eligible

88	Przybyłek	2019	Yes	Eligible
89	Valero	2020	Yes	Eligible
90	Choi	2015	Yes	Eligible
91	Lukanc	2020	Yes	Eligible
92	Vogt	2021	Yes	Eligible
93	Kasiotis	2023	Yes	Eligible
94	Wang	2022	Yes	Eligible
95	Nabi	2023	Yes	Eligible
96	Nabi	2022	Yes	Eligible

Rizzo, A., Piccinno, M., Lillo, E., Carbonari, A., Jirillo, F., & Sciorsci, R. L. Antimicrobial Resistance and Current A
Wang, J., Deng, L., Chen, M., Che, Y., Li, L., Zhu, L., Chen, G., & Feng, T. Phytogenic feed additives as natural an
Salman, M.D.; Rao, S.; Akbar, A.; Bahadur, S.U.K.; Heilmann, M.; Song, J. Alternatives to Antibiotic Growth Pro
Marshall, B. M., & Levy, S. B. Food animals and antimicrobials: impacts on human health. Clinical microbiolog
Landers, T. F., Cohen, B., Wittum, T. E., & Larson, E. L. A review of antibiotic use in food animals: perspective,
Ventola, C.L. The antibiotic resistance crisis. Pharm. Ther. 2015, 40, 277–283.
Matheou, A.; Abousetta, A.; Pascoe, A.P.; Papakostopoulos, D.; Charalambous, L.; Panagi, S.; Panagiotou, S.; Y
FAO. Antimicrobial Resistance in Livestock; FAO: Rome, Italy, 2015.
Mackenzie, J.S.; Jeggo, M. The One Health approach. Trop. Med. Infect. Dis. 2019, 4, 88. https://doi.org/10.33
Prestinaci, F.; Pezzotti, P.; Pantosti, A. Antimicrobial resistance overview. Pathog. Glob. Health 2020, 114, 1–10
WHO (World Health Organization). Global Antimicrobial Resistance Report; Geneva, Switzerland, 2023.
Ekor, M. The growing impact of antimicrobial resistance in veterinary medicine. Front. Microbiol. 2014, 5, 152
Laurino, C.; Palmieri, B. Zeolite: The magic stone. Rev. Environ. Sci. Biotechnol. 2015, 14, 341–358.
Oggiano, G., Pokimica, B., Popović, T., & Takić, M. Beneficial properties of zeolite. Italian Journal of Food Scien
Alotaibi, A.M. An Overview of Zeolites: From Historical Background to Diverse Applications. Molecules 2025, 30
Đuričić, D.; Beer Ljubić, B.; Vince, S.; Turk, R.; Valpotić, H.; Žura Žaja, I.; Maćešić, N.; Benić, M.; Getz, I.; Samardži
Folnožić, I.; Samardžija, M.; Đuričić, D.; Vince, S.; Perkov, S.; et al. Effects of clinoptilolite on metabolic and antic
Vince, S.; Večkovec, A.M.; Valpotić, H.; Špoljarić, D.; Žura Žaja, I.; Đuričić, D.; Leiner, D.; Šavorić, J.; Butković, I.; H
Maity, S.; Rubić, I.; Kuleš, J.; Horvatić, A.; Đuričić, D.; Samardžija, M.; Beer Ljubić, B.; Turk, R.; Gračner, D.; Maćeš
Pavelić, K., Hadzija, M., Bedrica, L., Pavelić, J., Dikić, I., Katić, M., Kralj, M., Bosnar, M. H., Kapitanović, S., Poljak-B
Valpotić, H.; Gračner, D.; Turk, R.; Đuričić, D.; Vince, S.; Folnožić, I.; Lojkić, M.; Žura Žaja, I.; Bedrica, L.; Maćešić,
Katsoulos, P.D.; Karatzia, M.A.; Boscos, C.; Wolf, P. & Karatzias, H. In-field evaluation of clinoptilolite feeding e
Valpotić, H.; Terzić, S.; Vince, S.; Samardžija, M.; Turk, R.; Lacković, G.; Habrun, B.; Đuričić, D.; Sadiković, M.; Va
McCollum F. T., Galyean M. L. Effects of Clinoptilolite on Rumen Fermentation, Digestion and Feedlot Perform
Amanzougarene, Z., & Fondevila, M. Rumen Fermentation of Feed Mixtures Supplemented with Clay Minerals
Tánori-Lozano, A.; López-Baca, M.Á.; Muhlia-Almazán, A.; Montalvo-Corral, M.; Pinelli-Saavedra, A.; Islava-Lag
Travagli, V., Zanardi, I., Valacchi, G., & Bocci, V. (2010). Ozone and ozonated oils in skin diseases: a review. Me
Bocci, V. Ozone: A New Medical Drug; Springer: Dordrecht, The Netherlands, 2020. Abd El-Aziz, A.; Abo Ghani
29. Banerjee B, Thompson C, Nizet V and Bjånes E. (2024) Bactericidal efficacy of low dose gaseous ozone agai
30. Sciorsci, R.L.; Lillo, E.; Occhiogrosso, L.; Rizzo, A. Ozone therapy in veterinary medicine. Res. Vet. Sci. 2020,
31. de Souza AKL, Colares RR, de Souza ACL. The main uses of ozone therapy in diseases of large animals: A rev
32. Đuričić, D.; Vince, S.; Ablondi, M.; Dobranić, T.; Samardžija, M. Effect of preventive intrauterine ozone appl
33. Scrollavezza, Paolo; Ablondi, M.; Pogliacomi, B.; Dall'Aglio, R.; Poldi, R.; Pezzoli, G. Ozone treatment in mas
34. Đuričić, D.; Valpotić, H.; Samardžija, M. Prophylaxis and therapeutic potential of ozone in buiatrics: Current
35. Đuričić, D.; Benić, M.; Maćešić, N.; Turk, R.; Cvetnić, L.; Gračner, D.; Dobranić, V.; Getz, I.; Lojkić, M.; Samar
36. Zobel R, Tkalčić S, Stoković I, Pipal I, Buić V. Efficacy of ozone as a novel treatment option for urovagina in c
37. Đuričić, D.; Samardžija, M.; Valpotić, H.; Žura Žaja, I. Comparison of intrauterine antibiotics versus ozone m
38. Đuričić, D.; Valpotić, H.; Samardžija, M. The intrauterine treatment of retained foetal membrane in dairy g
39. Đuričić, D.; Dobranić, T.; Vince, S.; Getz, I.; Gračner, D.; Grizelj, J.; Prvanović, N.; Folnožić, I.; Smolec, O.; Sar
40. Đuričić, D.; Vince, S.; Lojkić, M.; Jelušić, S.; Turk, R.; Valpotić, H.; Gračner, D.; Maćešić, N.; Folnožić, I.; Šosta
41. Elvis, A.M.; Ekta, J.S. Ozone therapy: A clinical review. J. Nat. Sci. Biol. Med. 2011, 2, 66–70.
42. Orlandin. JR, Machado LC, Ambrósio CE, Travagli V. Ozone and its derivatives in veterinary medicine: A car
43. Di Paolo, N.; Bocci, V.; Travagli, V. Ozone therapy in veterinary medicine: Applications and limitations. Vet.
44. Rubin, J., & Roman, M. Veterinary Medical Ozone Therapy: An Integrative Approach. The Veterinary clinics
45. Abu-Seida, A.M. Potential benefits of propolis in large and small animal practices: A narrative review of the
46. Bačić, G.; Maćešić, N.; Radin, L.; Aladrović, J.; Matanović, K.; Mašek, T.; Brozić, D.; Benić, M.; Radić, B.; Bači
47. Šuran J, Aladrović J, Ljubić BB, Vlainić J, Mamić M, Radić B, Bačić G, Mačešić N, Benić M, Kostelić A, et al. Th

48. Bava, R.; Puteo, C.; Lombardi, R.; Garcea, G.; Lupia, C.; Spano, A.; Liguori, G.; Palma, E.; Britti, D.; Castagna,
49. Özdemir V, Yanar M, Koçyiğit R. General Properties of Propolis and its Usage in Ruminants. Journal of the H
50. Abd El-Aziz, A.; Abo Ghanima, M.; Mota-Rojas, D.; Sherasiya, A.; Ciani, F.; El-Sabrout, K. Bee Products for Pe
51. Kuralkar, P.; Kuralkar, S.V. Role of herbal products in animal production. J. Ethnopharmacol. 2021, 278, 114
52. Yang, C.; Chowdhury, M.A.K.; Huo, Y.; Gong, J. Phytogenic Compounds as Alternatives to In-Feed Antibiotic
53. Pérez-Flores, J.G.; García-Curiel, L.; Pérez-Escalante, E.; Contreras-López, E.; Aguilar-Lira, G.Y.; Ángel-Jijón, C
54. Vercelli, C.; Amadori, M.; Gambino, G.; et al. Natural antimicrobial compounds in veterinary medicine. App
55. Alagawany, M.; Abd El-Hack, M.E.; Farag, M.R.; Tiwari, R.; Dhama, K.; Arain, M.A. Phytogenic feed additives
56. Benchaar C., Calsamiglia S., Chaves A.V., Fraser G.R., Colombatto D., McAllister T.A., Beauchemin, K.A. A re
57. Yang, F., Yang, F., Zhai, Z.-H., Wang, S.-Q., Zhao, L., Zhang, B.-L., Chen, J.-C., & Wang, Y.-Q. Effects of alfalfa
58. Kholif A. E. A Review of Effect of Saponins on Ruminal Fermentation, Health and Performance of Ruminant
59. Ramdani, D.; Yuniarti, E.; Jayanegara, A.; Chaudhry, A.S. Roles of Essential Oils, Polyphenols, and Saponins
60. Priyashantha, H., Jayathissa, I. S., Vidanarachchi, J. K., Jayarathna, S., Mapiye, C., Maggiolino, A., & Ponnam
61. Quintavalla, F. Phytotherapeutic approaches in canine pediatrics. Veterinary Sciences, 2024, 11(3), 133. ht
62. Tamminen, L. M., Emanuelson, U., & Blanco-Penedo, I. Systematic Review of Phytotherapeutic Treatments
63. Abo-El-Sooud K. Ethnoveterinary perspectives and promising future. International journal of veterinary sci
64. Alasbahi, R.H., & Groot, M. J. Ethnoveterinary uses of certain Yemeni plants: A review of the scientific evid
65. Oliveira, M., Hoste, H., & Custódio, L. A systematic review on the ethnoveterinary uses of mediterranean s
66. Mayer, M., Vogl, C. R., Amorena, M., Hamburger, M., & Walkenhorst, M. Treatment of organic livestock wi
67. Aziz, M.A., Khan, A.H. & Pieroni, A. Ethnoveterinary plants of Pakistan: a review. J Ethnobiology Ethnomedi
68. Dong, Z.; Chen, H.; Liu, Y. Macleaya cordata alkaloids reduce inflammatory responses in livestock. Front. Ve
69. Schlittenlacher, T., Knubben-Schweizer, G., Dal Cero, M., Vogl, C. R., Maeschli, A., Hamburger, M., & Walke
70. Holzner, L. A., Hamburger, M., Dal Cero, M., Maeschli, A., Vogl, C. R., Meier, B., Walkenhorst, M., & Schlitte
71. Oda, B.K., Lulekal, E., Warkineh, B., Asfaw Z, & Debella A. Ethnoveterinary medicinal plants and their utiliza
72. Wendimu, A.; Bojago, E.; Abrham, Y. Medicinal ethnoveterinary plants used for treating livestock ailments
73. Đurić Jarić, M.; Gottstein, Ž.; Vince, S.; Žura Žaja, I.; Brus, M.; Đuričić, D.; Samardžija, M.; Valpotić, H. Effect
74. Anywar, G.; Ssegabo, A.; Wanyama, J.; Weckerle, C.S. A review of ethnoveterinary botanical medicines use
75. Folnožić, I.; Đuričić, D.; Žura Žaja, I.; Vince, S.; Perkov, S.; Turk, R.; Valpotić, H.; Gračner, D.; Maćešić, N.; Loj
76. Samardžija, M.; Turk, R.; Sobiech, P.; Valpotić, H.; Harapin, I.; Gračner, D.; Đuričić, D. Intrauterine ozone tre
77. Bosi, P., Creston, D., & Casini, L. Production performance of dairy cows after the dietary addition of clinopt
78. Samardžija, M.; Folnožić, I.; Perkov, S.; et al. Influence of clinoptilolite on reproduction in dairy cows. Proc.
79. Samardžija, M.; Kowalczyk, A.; Kovačić, M.; Đuričić, D. Current knowledge of alternative therapy with ozon
80. Carbonari, A., Burgio, M., Frattina, L., Cicirelli, V., Iarussi, F., Tempesta, M., Lucente, M.S., Rizzo, A., & Grec
81. Escandón, B.M., Espinoza, J.S., Perea, F.P., Quito F., Ochoa R., López G. E., Galarza D.A. ,& Garzón J.P. Intra
82. Tomanić, D.; Samardžija, M.; Kovačević, Z. Alternatives to Antimicrobial Treatment in Bovine Mastitis Thera
83. Moroni, R., Fanelli, D., Maltinti, S., Orefice, M., Rota, A., Camillo, F., Mélanie, P., Cantile, C., Miragliotta, V.,
84. Melanie P, Niola C, Plataroti I, Mancini S, Fratini F. Use of Ozone in Veterinary Dentistry as an Alternative t
85. Chuttong, B.; Kumsaiyai, W.; et al. Functional properties and antimicrobial effects of propolis. Foods 2023,
86. da Silva, V. F., Rosa, D. S., de Figueirêdo, P. I., da Silva, T. M. S., Peixoto, R. M., & da Costa, M. M. Antimicro
87. Mikniene Z, Puska G, Liaudanskas M, Siugzdaite J, Kubiliene L, Rudejeviene J, Zvikas V, Sutkeviciene N, Raga
88. Wang, K.; Jin, X.L.; Shen, X.G.; Sun, L.P.; Wu, L.M.; Wei, J.Q.; Marcucci, M.C.; Hu, F.L.; Liu, J.X. Effects of Chi
89. Bischofberger, A.S.; Dart, C.M.; Horadagoda, N.; Perkins, N.R.; Jeffcott, L.B.; Little, C.B.; Dart, A.J. Effect of M
90. Chatzimisios, K.; Tsioli, V.; Brellou, G.D.; Apostolopoulou, E.P.; Angelou, V.; Pratsinakis, E.D.; Cremers, N.A.
91. Abdelnour, S.A.; Abd El-Hack, M.E.; Alagawany, M.; Farag, M.R.; Elnesr, S.S. Beneficial impacts of bee polle
92. Han, S.M.; Lee, K.G.; Yeo, J.H.; Oh, B.Y.; Kim, B.S.; Lee, W.; Baek, H.J.; Kim, S.T.; Pak, S.C. Effects of honeybe
93. Elkomy, A.; El-Hanoun, A.; Abdella, M.; El-Sabrout, K. Improving the reproductive, immunity and health sta
94. Aguiar, S.C.D., Paula, E.M.D., Yoshimura, E.H., Santos, W.B.R.D., Machado, E., Valero, M.V., Santos, G.T.D. a

95. Santos, L.M., Fonseca, M.S., Sokolonski, A.R., Deegan, K.R., Araújo, R.P., Umsza-Guez, M.A., Barbosa, J.D., F
96. Stevanović, J.; Glavinić, U.; Ristanić, M.; Erjavec, V.; Denk, B.; Dolašević, S.; Stanimirović, Z. Bee-Inspired He
97. Wagh, V.D. Propolis: A Wonder Bees Product and Its Pharmacological Potentials. Advances in Pharmacolog
98. Bankova, V.; Popova, M.; Trusheva, B. Propolis: Recent advances in chemistry and plant origin. Apidologie
99. Zulhendri, F.; Chandrasekaran, K.; Kowacz, M.; Ravalia, M.; Kripal, K.; Fearnley, J.; Perera, C.O. Antiviral, An
100. Maestrini, M., Tava, A., Mancini, S., Tedesco, D., & Perrucci, S. In vitro anthelmintic activity of saponins fro
101. Saeed, M.; Babazadeh, D.; Arif, M.; Arain, M.; Bhutto, Z.; Shar, A.; et al. Silymarin: a potent hepatoprotecti
102. Gutiérrez-Grijalva, E.P.; et al. Flavonoids and their antimicrobial activity. Food Sci. Nutr. 2020, 8, 1–12. http
103. Przybyłek, I.M.; Karpinski, T.M. Antibacterial Properties of Propolis. Molecules, 2019, 24, 2047.
104. Valero, M.S.; González, M.; Ramón-Gimenez, M.; Andrade, P.B.; Moreo, E.; Les, F.; et al. Jasonia glutinosa (
105. Choi, J.H.; Jang, A.Y.; Lin, S.; Lim, S.; Kim, D.; Park, K.; Han, S.M.; Yeo, J.H.; Seo, H.S. Melittin, a honeybee ve
106. Lukanc, B.; Potokar, T.; Erjavec, V. Complete skin regeneration with medical honey after skin loss on the er
107. Vogt, N.A.; Vriezen, E.; Nwosu, A.; Sargeant, J.M. A scoping review of the evidence for the medicinal use of
108. Kasiotis, K.M.; Zafeiraki, E.; Manea-Karga, E.; Anastasiadou, P.; Machera, K. Pesticide residues and metabo
109. Wang, X.; Wang, Y.; Mao, Y.; Hu, A.; Xu, T.; Yang, Y.; et al. Corrigendum: The beneficial effects of traditiona
110. Groot MJ, Berendsen BJA and Cleton NB. The Next Step to Further Decrease Veterinary Antibiotic Applicat
111. Nabi, F., Shi, D., Wu, Q., & Baloch, D. M. Editorial: Treatment of animal diseases with veterinary phytother
112. Nabi, F.; Arain, M.A. Rising stars in comparative and clinical medicine: 2021. Front. Vet. Sci. 2022, 9, 10309
113. Setayesh, M.; Karimi, M.; Zargaran, A.; Abousaidi, H.; Shahesmaeili, A.; Amiri, F.; et al. Efficacy of a Persian
1. McCorkle, C.M.; Martin, M. Parallels and potentials in animal and human ethnomedical techniques. Agric.
2. McCorkle, C.M. An introduction to ethnoveterinary research and development. J. Ethnobiol. 1986, 6, 129-
3. Drell, J.R.R. Neanderthals: A history of interpretation. Oxf. J. Archaeol. 2000, 19, 1–24. https://doi.org/10.
4. Spikins, P.; Needham, A.; Wright, B.; Dytham, C.; Gatta, M.; Hitchens, G. Neanderthal healthcare. Quat. Sc
5. El Zaatari, S.; Hublin, J.-J. Diet of Upper Paleolithic modern humans. Am. J. Phys. Anthropol. 2014, 153, 57
6. Krueger, K.L. Dental microwear analysis of the Krapina Neandertals. Cent. Eur. J. Geosci. 2012, 4, 651–652
7. Hoffecker, J.F. The spread of modern humans in Europe. Proc. Natl. Acad. Sci. USA 2009, 106, 16040–160
8. Cooper, M.T. The roles of nature, deities, and ancestors in constructing religious identity in contemporary
9. El-Soud, N.H.A. Herbal medicine in ancient Egypt. J. Med. Plant Res. 2010, 4, 82–86.
10. Manniche, L. An Ancient Egyptian Herbal; University of Texas Press: Austin, TX, USA, 1989; p. 172.
11. Farnell, L.R. Greek Hero Cults and Ideas of Immortality; Clarendon Press: Oxford, UK, 1921; Chapter 10, "T
12. Dugački, V. Healthcare between East and West: Croatia at the crossroads of European medical traditions.
13. Jacques, J. Hippocrates, 2nd ed.; Johns Hopkins University Press: Baltimore, MD, USA, 2001; p. 536.
14. Modrak, D.K.W. Theophrastus and Recent Scholarship. J. Hist. Ideas, 1994, 55, 337-345. https://doi.org/1
15. De Vos, P. European materia medica in historical texts: Longevity of a tradition. J. Ethnopharmacol. 2010,
16. Sadek, M.M. The Arabic Materia Medica of Dioscorides; Les Éditions du Sphinx: Québec, Canada, 1983.
17. Lazris, J.; Stavros, V. Anatomical schemata from Aristotle to Dioscorides. Pallas 2013, 93, 131–164. https:/
18. Doyen-Higuet, A.-M. The Hippiatrica and Byzantine veterinary medicine. Dumbarton Oaks Pap. 1984, 38,
19. Oparin, O.A. Medicine in the Byzantine Empire: History and philosophy. Shidnoevr. Z. Vnutr. Simejnoi Me
20. Mayer, M.; Vogl, C.R.; Amorena, M.; Hamburger, M.; Walkenhorst, M. Treatment of Organic Livestock wit
21. Mayer, M.; Zbinden, M.; Vogl, C.R.; Ivemeyer, S.; Meier, B.; Amorena, M.; Maeschli, A.; Hamburger, M.; W
22. Martin, M.; Mathias, E.; McCorkle, C.M. Ethnoveterinary Medicine: An Annotated Bibliography of Commu
23. Franz, C. Herbs and Herbal Products in Animal Nutrition and Veterinary Medicine. Afr. J. Tradit. Compleme
24. Đuričić, D. Livestock organic production and ethnoveterinary practice in Europe. In Integration of Educatio
25. Đuričić, D.; Samardžija, M. Traditional ethnoveterinary knowledge of indigestion and diarrhoea treatment
26. Zitterl-Eglseer, K.; Moder, H.; Franz, C.; Zitterl, W. Phytopharmaceuticals for sheep and goats. Wien. Tierä
27. Zitterl-Eglseer, K.; Ludwig, M.; Franz, C. Phytotherapy in cattle: Historical and modern perspectives. Wien.
28. Bullitta, S.; Re, G.A.; Manunta, M.D.I.; Piluzza, G. Traditional knowledge about plant, animal, and mineral-

29. Guarrera PM. Traditional antihelmintic, antiparasitic and repellent uses of plants in Central Italy. J Ethnop
30. Birrenkott, G.P.; Brockenfelt, G.E.; Greer, J.A.; Owens, M.D. Topical application of garlic reduces northern
31. Perrucci, S.; Flamini, G.; Cioni, P.L.; Morelli, I.; Macchioni, F.; Macchioni, G. Efficacy of Artemisia verlotoru
32. Waller, P.J.; Bernes, G.; Thamsborg, S.M.; Sukura, A.; Richter, S.H.; Ingebrigsen, K.; Höglund, J. Plants as de
33. Zenner, L.; Callait, M.P.; Granier, C.; Chauve, C. Essential oils against poultry protozoa. Parasite 2003, 10,
34. Vitalini, S.; Iriti, M.; Puricelli, C.; Ciuchi, D.; Segale, A.; Fico, G. Alpine ethnobotanical study in Val San Giac
35. Pieroni, A.; Giusti, M.E.; Münz, H.; et al. Ethnobotanical knowledge of the Istro-Romanians of Zejane (Croa
36. Pieroni, A.; Quave, C.L.; Nebel, S.; Heinrich, M. Ethnopharmacy of the Arbëreshë in southern Italy. Fitoter
37. Agelet, A.; Vallès, J. Vascular plants used in ethnoveterinary medicine in Pallars (Pyrenees, Catalonia, Iber
38. Carrió, E.; Rigat, M.; Garantje, T.; Mayans, M.; Parada, M.; Vallès, J. Plant ethnoveterinary practices in Cat
39. Bartha, S.G.; Quave, C.L.; Balogh, L.; Papp, N. Ethnoveterinary practices of Covasna County, Transylvania,
40. Lentini, F.; Raimondo, F.M. Indagini etnobotaniche in Sicilia. IV. L'uso popolare delle piante nel territorio d
41. Mattalia, G.; Belichenko, O.; Kalle, R.; Kolosova, V.; Kuznetsova, N.; Prakofjewa, J.; Stryamets, N.; Pieroni,
42. Brag, S.; Hansen, H.-J. Treatment of ruminal indigestion according to popular belief in Sweden. Rev. Sci. T
43. Van Asseldonk, T.; Beijer, H. Herbal folk remedies for animal health in the Netherlands. In Proc. 4th Int. C
44. Blanco, E.; Macía, M.J.; Morales, R. Medicinal and veterinary plants of El Caurel (Galicia, NW Spain). J. Eth
45. Gonzalez JA, Garcia-Barriuso M, Amich F. Ethnobotanical study of medicinal plants traditionally used in th
46. Blanco, E.; Macía, M.J.; Morales, R. Unspecific stimulation therapy by transcutaneous implantation of Hel
47. Benítez, G.; Reyes González-Tejero, M.; Molero-Mesa, J. Knowledge of ethnoveterinary medicine in the P
48. González-Tejero, M.R.; Molero-Mesa, J.; Martínez-Cobo, A.; Guzmán, A.; El-Ouardani, F. Medicinal plants
49. Farinha, N.; Póvoa, O.; Santos, R. Ethnoveterinary applied to Equidae in the Alentejo, south Portugal. In b
50. Lentini, F. Indagini etnobotaniche in Sicilia. II. L'uso tradizionale delle piante in alcune comunità del Trapa
51. Lentini, F.; Aleo, M. Indagini etnobotaniche in Sicilia. V. L'uso tradizionale delle piante nel territorio di Eric
52. Lentini, F.; Catanzaro, F.; Aleo, M. Indagini etnobotaniche in Sicilia. III. L'uso tradizionale delle piante nel t
53. Amico, F.P.; Sorge, E.G. Medicinal plants and phytotherapy in the Mussomeli area (Caltanissetta, Sicily, Ita
54. Barbagallo, C.; Furnari, F. Flora Officinale del Territorio di Caltagirone (Catania); Editrice Fusi: Pavia, Italy,
55. Barbagallo, C.; Grillo, M.; Meli, R. Nota sulle piante officinali spontanee e coltivate del territorio di Cesarò
56. De Feo, V.; Aquino, R.; Menghini, A.; Ramundo, E.; Senatore, F. Traditional phytotherapy in the Sorrentina
57. De Feo, V.; Senatore, F. Medicinal plants and phytotherapy in the Amalfitan Coast (Campania, southern It
58. Leporatti, M.L.; Pavesi, A. New, rare or interesting uses of officinal plants in Calabria. Webbia 1989, 43, 2
59. Pieroni, A.; Howard, P.; Volpato, G.; Santoro, R.F. Natural remedies and nutraceuticals in southern Italy. V
60. Bullitta, S.; Piluzza, G.; Viegi, L. Plant resources used for traditional ethnoveterinary phytotherapy in Sardi
61. Guarrera, P.M.; Forti, G.; Marignoli, S. Ethnobotanical and ethnomedicinal uses of plants in central Italy. J
62. Guarrera, P.M.; Tammaro, F.; Bernardini, D.; Carluccio, A. Ethnoveterinary phytotherapy in central Italy. I
63. De Simoni, E.; Guarrera, P.M. Indagine etnobotanica nella Provincia di Teramo. Quad. Bot. Ambient. Appl.
64. Uncini Manganelli, R.E.; Camangi, F.; Tomei, P.E. Curing animals with plants in Tuscany. J. Ethnopharmaco
65. Viegi, L.; Bioli, A.; Vangelisti, R.; Cela Renzoni, G. Veterinary folk medicine in Alta Val di Cecina. Atti Soc. T
66. Pieroni, A.; Giusti, M.E. Ritual botanicals against the evil eye in Tuscany. Econ. Bot. 2002, 56, 201–204.
67. Erciyes, C.Ç.S.; Yaşar, A. The Use of Herbal Drugs in Organic Animal Production: The Case of Ethnoveterina
68. Ertuğ, F. Plant, animal and human relationships in Turkish folk medicine. In Herbs, Humans and Animals; F
69. Zouraris, D.; Graikou, K.; Vasileiou, P.; Dimitrov, V.; Dajic Stevanovic, Z.; Bilia, A.R.; Zivkovic, J.; Dias, A.; Ka
70. Vucevat-Bajt, V.; Karlović, M. Traditional treatment of animal diseases in Croatia. Rev. Sci. Tech. OIE 1994
71. Janaćković, P.; Gavrilović, M.; Miletić, M.; Radulović, M.; Kolašinac, S.; Stevanović, Z.D. Small regions as ke
72. Pieroni A, Dibra B, Grishaj G, Grishaj I, Gjon Maçai S. Traditional phytotherapy of the Albanians of Lepushe
73. Šubarević, N.; Stevanović, O.; Petrujkić, B. Use of phytotherapy as a form of ethnoveterinary medicine in
74. Marković, M.S.; Pljevljakušić, D.S.; Nikolić, B.M.; Miladinović, D.L.; Djokić, M.M.; Rakonjac, L.B.; Jovanović,
75. Rexhepi, B.; Behxhet, M.; Hajdari, A.; Rushidi-Rexhepi, J.; Quave, C.L.; Pieroni, A. Traditional medicinal pla

76. Casaru, C.; Bulgaru, A.; Danes, D. Medicinal plants used in traditional veterinary medicine to treat rumina
77. Boycheva, P.; Zahariev, D. Use Of Medicinal Plants In The Veterinary Medicine In The Northern Black Sea
78. Stucki, K.; Cero, M.D.; Vogl, C.R.; Ivemeyer, S.; Meier, B.; Maeschli, A.; Hamburger, M.; Walkenhorst, M. E
79. Mertenat, D.; Cero, M.D.; Vogl, C.R.; Ivemeyer, S.; Meier, B.; Maeschli, A.; Hamburger, M.; Walkenhorst, M
80. Schlittenlacher, T.; Knubben-Schweizer, G.; Dal Cero, M.; Vogl, C.R.; Maeschli, A.; Hamburger, M.; Walken
81. Schlittenlacher, T.; Knubben-Schweizer, G.; Dal Cero, M.; Vogl, C.R.; Maeschli, A.; Hamburger, M.; Walken
82. Korpelainen, H.; Pietiläinen, M. What Makes a Good Plant Invader? Life 2023, 13, 1596. https://doi.org/1
83. Bobuská, L.; Demková, L.; Pinčáková, G.; Lošák, T. Alien Plant Invasion: Are They Strictly Nature's Enemy a
1. Samardžija, Marko; Kowalczyk, Alicja; Kovačić, Mislav; Đuričić, Dražen Current knowledge of alternative thera
2. Samardžija, Marko ; Turk, Romana ; Sobiech, Przemyslaw ; Valpotić, Hrvoje ; Harapin, Ivica ; Gračner,
3. Đuričić, Dražen ; Valpotić, Hrvoje ; Žura Žaja, Ivona ; Samardžija, Marko Comparison of intrauterine a
4. Samardžija, Marko ; Valpotić, Hrvoje ; Đuričić, Dražen ; Szenci, Ottó Intrauterine use of ozone in dom
5. Đuričić, Dražen ; Samardžija, Marko EFFICACY OF OZONE AS A NOVEL THERAPEUTIC AGENT FOR END
6. Đuričić, Dražen ; Dobranić, Tomislav ; Valpotić, Hrvoje ; Folnožić, Ivan ; Harapin, Ivica ; Samardžija, M
7. Đuričić, Dražen ; Valpotić, Hrvoje ; Samardžija, Marko Prophylaxis and therapeutic potential of ozone
8. Đuričić, Dražen ; Valpotić, Hrvoje ; Samardžija, Marko The Intrauterine Treatment of the Retained Fo
9. Samardžija, Marko ; Dobranić, Tomislav ; Prvanović Babić, Nikica ; Lojkić, Martina ; Folnožić, Ivan ; Đu
10. Švaganović, Lena Iva UTJECAJ PREVENTIVNE INTRAUTERINE UPOTREBE OZONA NA RASPLODNU SPOS
11. Đuričić, Dražen ; Lipar, Marija ; Samardžija, Marko Ozone treatment of metritis and endometritis in H
12. Zobel, Robert ; Martinec, Robert ; Ivanović, Duško ; Rošić, Nikola ; Stančić, Zdravka ; Žerjavić, Ivica ; F
13. Đuričić, Dražen ; Ablondi, Marco ; Samardžija, Marko ; Tomica, Darko ; Herceg, Željko ; Dobranić, Ton
14. Đuričić, Dražen ; Samardžija, Marko PRIMENA OZONA U POBOLJŠANJU REPRODUKTIVNE EFIKASNOST
15. Đuričić, Dražen ; Vince, Silvijo ; Ablondi, Marco ; Dobranić, Tomislav ; Samardžija, Marko Intrauterine
16. Zobel, Robert ; Tkalčić, Suzana ; Štoković, Igor ; Pipal, Ivana ; Buić, Vlatka Efficacy of Ozone as a Nove
17. Đuričić, Dražen ; Vince, Silvijo ; Ablondi, Marco ; Dobranić, Tomislav ; Samardžija, Marko Effect of Pre
18. Zobel, Robert ; Tkalčić, Suzana ; Štoković, Igor ; Smolec, Ozren ; Pipal, Ivana ; Buić, Vlatka Ozone as a
19. Đuričić, Dražen ; , Dobranić, Tomislav ; Vince, Silvijo ; Getz, Iva ; , Gračner, Damjan ; Grizelj, Juraj ; Prv
20. Tlak Gajger, Ivana Uspostavljanje sustava transpozon mutageneze za bakteriju Paenibacillus larvae /
21. Dovč, Alenka ; Vlahović, Ksenija ; Suhadolc-Scholten, Sara ; Tozon, Nataša Presence of ig G antibodies
22. Dovč, Alenka ; Vlahović, Ksenija ; Suhadolc, Sara ; Tozon, Nataša Seroprevalence of Chlamydophila fe
1. Maity, Sudipa ; Rubić, Ivana ; Kuleš, Josipa ; Horvatić, Anita ; Đuričić, Dražen ; Samardžija, Marko ; Beer Ljubić,
2. Šperanda, Tomislav ; Pavić, Valentina ; Lončarić, Zdenko ; Šperanda, Marcela ; Popović, Maja ; Gantn
3. Đuričić, Dražen ; Vince, Silvijo ; Lojkić, Martina ; Jelušić, Sanja ; Turk, Romana ; Valpotić, Hrvoje ; Grač
4. Đuričić, Dražen ; Sukalić, Tomislav ; Marković, Franjo ; Kočila, Predrag ; Žura Žaja, Ivona ; Menčik, Sve
5. Đuričić, Dražen ; Beer Ljubić, Blanka ; Vince, Silvijo ; Turk, Romana ; Valpotić, Hrvoje ; Žura Žaja, Ivona
6. Folnožić, Ivan ; Samardžija, Marko ; Đuričić, Dražen ; Vince, Silvijo ; Perkov, Sonja ; Jelušić, Sanja ; Val
7. Folnožić, Ivan ; Đuričić, Dražen ; Žura Žaja, Ivona ; Vince, Silvijo ; Perkov, Sonja ; Turk, Romana ; Valpo
8. Đuričić, Dražen ; Turk, Romana ; Lojkić, Martina ; Jelušić, Sonja ; Valpotić, Hrvoje ; Vince, Silvijo ; Foln
9. Đuričić, Dražen ; Turk, Romana ; Lojkić, Martina ; Jelušić, Sanja ; Valpotić, Hrvoje ; Vince, Silvijo ; Foln
10. Đuričić, Dražen ; Benić, Miroslav ; Maćešić, Nino ; Turk, Romana ; Cvetnić, Luka ; Gračner, Damjan ; D
11. Turk, Romana ; Đuričić, Dražen ; Vince, Silvijo ; Grizelj, Juraj ; Flegar-Meštrić, Zlata ; Perkov, Sonja ; Je
12. Đuričić, Dražen ; Turk Romana ; Beer-Ljubić, Blanka ; Valpotić, Hrvoje ; Maćešić, Nino ; Benić, Mirosla
13. Valpotić, Hrvoje ; Žura Žaja, Ivona ; Samardžija, Marko ; Habrun, Boris ; Ostović, Mario ; Đuričić, Draž
14. Samardžija, Marko ; Đuričić, Dražen ; Benić, Miroslav ; Maćešić, Nino ; Valpotić, Hrvoje ; Gračner, Dar
15. Valpotić, Hrvoje ; Gračner, Damjan ; Turk, Romana ; Đuričić, Dražen ; Vince, Silvijo ; Folnožić, Ivan ; Lo
16. Đuričić, Dražen ; Benić, Miroslav ; Maćešić, Nino ; Valpotić, Hrvoje ; Turk, Romana ; Dobranić, Vesna ;
17. Valpotić, Hrvoje ; Barić-Rafaj, Renata ; Mrljak, Vladimir ; Grabarević, Željko ; Samardžija, Marko ; Špe

18. Samardžija, Marko ; Đuričić, Dražen ; Turk, Romana ; Valpotić, Hrvoje ; Gračner, Damjan ; Benić, Mirc
19. Valpotić, Hrvoje ; Terzić, Svjetlana ; Vince, Silvijo ; Samardžija, Marko ; Turk, Romana ; Lacković, Gord
20. Tlak Gajger, Ivana ; Ribarić, Jasna ; Matak, Marina ; Svečnjak, Lidija ; Kozarić, Zvonimir ; Nejedli, Srebr
21. Špiranec, Katarina ; Špoljarić, Daniel ; Mršić, Gordan ; Špoljarić, Igor ; Srčec, Siniša ; Cvrtila Fleck, Željk
22. Šperanda, Marcela ; Valpotić Hrvoje ; Šperanda, Tomislav ; Đidara, Mislav ; Antunović, Zvonko ; Miku
23. Hrenović, Jasna ; Tofant, Alenka ; Ostović, Mario ; Milić, Dinka Effect of clinoptilolite addition on bact
24. Machaček, Miroslav ; Večerek, Vladimir ; Mas, Nora ; Suchy, Pavel ; Strakova, Eva ; Šerman, Vlasta ; H
25. Straková, Eva ; Suchý, Pavel ; Herzig, Ivan ; Šerman, Vlasta ; Mas, Nora The long-term administration

Malatesta, Manuela, Gabriele Tabaracci, and Carlo Pellicciari. "Low-dose ozone as a eustress inducer

Wu, T., Liu, H., Xu, R., Li, Z., & Wei, Y. (2024). Differences in cellular and molecular processes in expos

Orlandin, J. R., Machado, L. C., Ambrósio, C. E., & Travagli, V. (2021). Ozone and its derivatives in vete

Slavinskienė, G., Grigonis, A., Ivaškienė, M., Sinkevičienė, I., Andrulevičiūtė, V., Ivanauskas, L., ... & Da

Kaneki, M., Ohira, C., Takahashi, M., Iwashita, N., Takagi, Y., Nagane, M., ... & Fukuyama, T. (2023). Tl

Repciuc, C. C., Vișan, G. A. M., Teleky, B. E., Pintea, A., Novac, C. Ș., & Oros, N. V. (2025). Physico-Che

Lillo, E., Cordisco, M., Trotta, A., Greco, G., Carbonari, A., Rizzo, A., ... & Corrente, M. (2023). Evaluati

Silva, L. P., Portela, R. W., Machado, M. C., Canuto, G. A. B., Costa-Neto, J. M., Carvalho, V. D. M. P. D

Dos Santos, T. G., Orlandin, J. R., de Almeida, M. F., Scassiotti, R. F., Oliveira, V. C., Santos, S. I. P., ... &

Machado, C. S., Seeger, M. G., Moreira, K. S., Burgo, T. A., Iglesias, B. A., Vogel, F. S., & Cargnelutti, J.

Donato, G. G., Nebbia, P., Stella, M. C., Scalas, D., Necchi, D., Bertero, A., ... & Nervo, T. (2024). In vitr

Sumida, J. M., Matera, J. M., & Hayashi, A. M. (2023). Randomized single-blinded prospective compa

Ferreira, J. C., Pires, R. H., da Costa, G. B., Carrijo, B. N., Guiotto, F. G., & Rodrigues, V. S. (2021). The i

Donato, G. G., Appino, S., Bertero, A., Poletto, M. L., Nebbia, P., Robino, P., ... & Nervo, T. (2023). Saf

Melanie, P., Niola, C., Plataroti, I., Mancini, S., & Fratini, F. (2024). Use of ozone in veterinary dentistr

Malatesta, M., & Carton, F. (2025). Nanocarriers for Medical Ozone Delivery: A New Therapeutic Stra

Carbonari, A., Burgio, M., Frattina, L., Cicirelli, V., & Rizzo, A. (2024). Repeat Breeder Syndrome Thera

Lillo, E., Pellegrini, F., Rizzo, A., Lanave, G., Zizzadoro, C., Cicirelli, V., ... & Camero, M. (2023). In vitro

Nguyen Ngoc, D., Valverde Piedra, J. L., Milczak, A., Szponder, T., Drzewiecka, B., Pyzerska, A., ... & W

Zatti, P. H., da Silva, N. P., Rigotti, M., Scariot, F. J., Davidson, C. B., Machado, A. K., & Branco, C. S. (2(

da Silva Rocha, M. R., Bueno, M. R., de Meira, W. J. T., Prestes, Y. S., & de Fátima Rodrigues, D. (2022

Carbonari, A., Burgio, M., Frattina, L., Cicirelli, V., Iarussi, F., Tempesta, M., ... & Greco, G. (2026). Intr

de Oliveira, V. A. P., Pinto, M. P. R., Larangeira, D. F., Silva, L. P., de Carvalho, V. D. M. P., Canuto, G. A

Aamer, H., Nour, E., Refaie, A., Youssef, M., & El-Ashker, M. (2023). Current perspectives for the use

Bungărdean, D., Pall, E., Daradics, Z., Popescu, M., Tripon, M. A., Lupșan, A. F., ... & Marcus, I. (2025).

Peteoacă, A., Istrate, A., Goanță, A. M., IONAȘCU, I., & Tănase, A. (2020). The use of ozone therapy in

Braga, C. Q., Zambrano, C. G., dos Santos Bermann, C., Milech, A., Ianiski, L. B., Soares, M. P., ... & Pe

Santos, J. K. V. R. S., Costa, E. D. D. S., Fernandes, C. C. L., Cortez, A. A., Fonsêca, A. D. V., Guedes, R. F

Remoli, G., Inguscio, C. R., Boschi, F., Tabaracci, G., Malatesta, M., & Cisterna, B. (2026). Low ozone c

dos Santos, J., de Souza, M. G., Barbosa, N. S. F., Bueno, R. D. C. L., & Fernandes, D. R. (2023). Uso da

Yñiguez, F. J. M., & Leise, B. S. (2025). Complications in Wound Management. Veterinary Clinics: Equi

de Luna Paiva, T. D., de Melo Lopes, A. R., Silva, Á. J. C., Figueiredo, M. A. F., da Cunha Nogueira, S. S.

Achy, J., Fuentes, E. A., Dos Santos, L. M., Neto, J. C. D. S., dos Santos Freire, C. C., Graboschii, A. C., ..

Rodrigues, V. S., Trevisan, L. A. C., Cintra, B. S., Pires, R. H., Ribeiro, A. B., Tavares, D. C., ... & Ferreira,

Morgoglione, M. E., Bosco, A., Ciuca, L., Pepe, P., Coles, G. C., Cringoli, G., & Rinaldi, L. (2021). In vitro

Souza, V. R., Carvalho, R. S., Coelho, C. S., & Dâmaso, Â. (2026). Clinical, haematological, and biochen

Ahmed, H. A., Hussein, A. E. D., El-Diasty, M., Likita, I. Y., Udechukwu, C. C., Jeremiah, O. T., ... & Ali, A

Krishnamoorthy, S., Moses, J. A., & Anandharamakrishnan, C. (2023). COVID-19, food safety, and con

DE CAMPOS, M., MARQUES, G., & TAVARES, D. (2023). O USO DA OZONIOTERAPIA NA CLÍNICA DE PE

Rafiei, Y., Ghamsari, S. M., Sasani, F., Ezzaty, A., Golchin, D., Akbari, A. M., & Fallah, A. (2025). Evaluat
Achar, P. N., Kritzinger, Q., & Sharma, S. (2024). Strategies for the Control of Aflatoxigenic Aspergillus
ASLAN, R. (2023). Holistic applications in veterinary medicine. BIDGE Publications.
Kayişoğlu, Ç., & Türksoy, S. (2023). OZON UYGULAMASININ TAHIL VE ÜRÜNLERİ ÜZERİNDEKİ ETKİLER
MIXIC, O., DE POLLO, D. E. Y. S., & GRANJA, I. I. E. U. (2023). Facultad de Medicina Veterinaria y Zoote
Матвеев, Д. В., Кошелева, И. В., Биткина, О. А., Сорокина, Т. В., Багателия, З. А., Долидзе, Д. Д., .
Carvalho, A. C., Silva, J. V. S., Lopes, J. W. C., Araújo, O. R. P., Goulart, M. O. F., Mariz, T. M. A., ... & Es
Iarussi, F., Paradiso, N., De Monte, V., Ferri, F., Ferulli, F., Caratozzolo, M. F., ... & Paradies, P. (2025).
Leiva, M., Vilao, R., Gaztelu, L., & Peña, T. (2026). Beyond Antibiotics: The Emerging Role of Antisepti
Chica, A. S., Álvarez, J. A. C., & Bossa, B. J. R. (2022). Efecto de la aplicación intravenosa de ozono en
Saleh, W., Khashjoori, B., Naeem, R., & Ibrahim, A. (2026). Preliminary study on ozone hemotherapy
Silva, A. B. P., & Yasuoka, M. M. Ozonioterapia na Prevenção e Terapêutica de Mastite em Vacas Leit
Campos Júnior, L. J., Guimarães, J. D., Gomez-Leon, V. H., & Freitas, B. W. D. (2023). Tratamentos alte
wasfi Sadeq, A. (2025). Histopathological evaluation of Utilizing decellularized xenograft cartilage and
de Souza Silva, M. E., de Freitas Magalhães, B., de Abreu, A. P. M., de Moraes, R. F. F., dos Santos Filh
Ільніцький, М. Г., Шаганенко, Р. В., & Шаганенко, В. С. (2024). Озонотерапія за лікування собак і
Sumida, J. M. (2021). Ozonioterapia versus eletroacupuntura como tratamento de cães com discopat
Martínez-Torrecilla, C., Cánovas-Ortiz, D., Priego-González, A., Fuertes-Recuero, M., Cerdeira-Lozano
Silva, K. A., & de Araujo Goes, P. A. Eficiência da Ozonioterapia Intra-Articular na Osteoartrite Canina
dos Santos, J., de Souza, M. G., Barbosa, N. S. F., Bueno, R. D. L., & Fernandes, D. R. (2023). The use o
AITALWAD, R., SAWALE, A., & MARKANDEYA, N. (2021). STUDIES ON OZONE AND AUTOLOGOUS PLA
Мочернюк, М. М., Кухтин, М. Д., Горюк, Ю. В., & Данилков, С. О. (2023). ЕФЕКТИВНІСТЬ ЗАСТОСУ
Aslan, N. E., & Erol, H. (2022). Medikal Ozonun Deri Lezyonlarının İyileşmesi Üzerine Etkileri. Bozok V
Vasconcelos, J. G., de Souza, M. E. M., de Andrade, A. B. P., Colares, J. C., Teles Filho, A. C. D. A., & Sa
de Oliveira, W. S., Inomata, S. B., Piveta, L. C., & dos Santos, J. P. Uso da ozonioterapia na buiatria.
Скляров, П. М. (2022). Development of protocols and efficiency of ozone-containing drugs for the tr
VÁZQUEZ ROMERO, Y. I. OZONOTERAPIA COMO TRATAMIENTO DE HERIDAS Y LESIONES DERMATOL
Aslan, N. E., & Erol, H. (2022). Effects of medical ozone on healing of skin lesions.
Rocha, M. R. D. S., Bueno, M. R., de Meira, W. J. T., Prestes, Y. S., & Rodrigues, D. D. F. (2022). Use of
Simões, A. C. G. (2021). Medicina Integrativa em clínica de animais de companhia.
Zakian, A., & Davoodi, F. (2020). A fresh look at the latest treatment methods of complementary and
Skliarov, P. M., Fedorenko, S. Y., Onyshchenko, O. V., Pasternak, A. M., Lieshchova, M. A., Bilyi, D. D.,
Đuričić, D., Valpotić, H., & Samardžija, M. (2015). Prophylaxis and therapeutic potential of ozone in b
Mylostyvyi, R. The effectiveness of ozone therapy in goats with mastitis.
Samardžija, M., Turk, R., Sobiech, P., Valpotić, H., Harapin, I., Gračner, D., & Đuričić, D. (2017). Intrau
Kerek, Á., Csanády, P., & Jerzsele, Á. (2022). Antibacterial efficiency of propolis-Part 1. Magyar Állato
Goswami, T. (2011). Ozone therapy: as an alternative medicine for bovine mastitis.
Özdemir, V. F., Yanar, M., & KOÇYİĞİT, R. (2022). General properties of propolis and its usage in rumi
Svetikiene, D., Zamokas, G., Jokubaite, M., Marksa, M., Ivanauskas, L., Babickaite, L., & Ramanauskie
Güzel, Ö., Yıldar, E., & Erdİkmen, D. O. (2011). Medical ozone and its use in veterinary surgery.
Adam, K., Peter, C., & Akos, J. (2022). Antiprotozoal and antifungal efficiency of propolis-Part 2. Magy
Kerek, Á., Csanády, P., Tuska-Szalay, B., Kovács, L., & Jerzsele, Á. (2023). In vitro efficacy of hungarian
Anghel, A. C., & Ţăranu, I. (2023). Antimicrobial potential of polyphenols obtained from agro-industri
Papaioannou, D. S., Kyriakis, S. C., Papasteriadis, A., Roumbies, N., Yannakopoulos, A., & Alexopoulos
Repciuc, C. C., Crecan, C. M., & Oană, L. I. (2016). Ozone therapy in veterinary medicine. Rev Room M
El-Badawi, A. Y., Yacout, M. H. M., Hafsa, S. H. A., & Hassan, A. A. (2015). Application of ozone treatm
Ádám, K., Péter, C., & Ákos, J. (2022). A propolisz protozoa-és gombaellenes hatékonysága-2. rész. M

Manav, S., Yilmaz, M., Baytekin, H., Çelik, K., & Çağli, A. (2020). The use of propolis as an antimicrobia
Higano, L. M., Kiefer, C., & Silva, C. M. (2020). Use of propolis for weaned piglets.
Kerek, Á., Yurt, A., Szabó, Á., Tuska-Szalay, B., & Jerzsele, Á. (2025). Antimicrobial resistance and natu
Solomakos, N., & Govaris, A. (2004). Oregano, thyme and sage, as natural additives to foods. Journal
Abdallah, M. S., Hassan, H. M., Ibrahim, W. A., Helal, A. M., & Hamed, E. A. (2023). Herbal oils and pr
Queiroga, M. C., Andrade, N., & Laranjo, M. (2018). Antimicrobial action of propolis extracts against s
FAtIMA, S., & BAYRAM, İ. (2024). tHE USE OF OREGANO IN SHEEP NUtRItION. Current Research in Ve
Kabakcı, D. (2022). An overview on the Effects of Propolis Administration in different branches of Liv
Papatsiros, V. G., Katsoulos, P. D., Koutoulis, K. C., Karatzia, M., Dedousi, A., & Christodoulopoulos, G
Valdenassi, L., Franzini, M., Garbelli, P., & Camolese, M. (2016). Oxygen-ozone activity in making fact
Jabbar, M., Hira, H., Saeed, M. S., Bibi, M., & Nawaz, H. (2022). The Propolis and Its Usability for the M
Bocci, V.; Travagli, V.; Zanardi, I. — *Ozone: A new medical drug* — 2011
Elvis, A. M.; Ekta, J. S. — *Ozone therapy: A clinical review* — 2011
Sechi, L. A.; Lezcano, I.; Nunez, N.; Espim, M.; Duprè, I.; Pinna, A.; Molicotti, P.; Fadda, G.; Zanetti, S. -
Haddad, M. A.; Souza, M. V.; Cecchini, A. L.; Moreira, J. J. — *Ozone therapy in veterinary medicine: A*
Martínez-Sánchez, G.; Al-Dalain, S. M.; Menéndez, S.; Re, L.; Giuliani, A.; Candelario-Jalil, E.; Alvarez, I
Zanardi, I.; Borrelli, E.; Valacchi, G.; Travagli, V.; Bocci, V. — *Ozone: A multifaceted molecule with une*
Sagai, M.; Bocci, V. — *Mechanisms of action involved in ozone therapy: Is healing induced via a mild o*
Akey, B.; Walton, T.; Rapp, D. — *Use of ozone therapy in canine wound management* — 2018
Ribeiro, A. M.; da Silva, R. S.; et al. — *Ozone therapy in veterinary medicine: Clinical applications* — 2
da Silva, R. S.; Cardoso, J. B.; et al. — *Ozone therapy in veterinary medicine: A review* — 2020
Aslaner, A.; et al. — *The main uses of ozone therapy in diseases of large animals: A review* — 2021
da Silva, R. S.; et al. — *Ozone therapy in veterinary medicine: Clinical indications and techniques* — 20
dos Santos, T. M.; et al. — *O uso da ozonioterapia na clínica de pequenos animais: revisão* — 2023
López, M. A.; et al. — *Ozone therapy by rectal insufflation in dogs: Safety and oxidative stress (randor*
Rossi, F.; et al. — *Preoperative and postoperative ozone therapy in cats undergoing mastectomy: A sh*
Bianchi, C.; et al. — *Integrative therapies in wound healing in small animals* — 2026
Menéndez, S.; Falcón, L.; Maqueira, Y. — Therapeutic efficacy of ozone in animals with parvoviral inf
Díaz-Luis, J.; Menéndez, S.; et al. — Ozone therapy in veterinary medicine: Applications in infectious
Restrepo, L.; Díaz, M.; et al. — Use of ozone therapy in bovine mastitis — 2013
Ogata, A.; Nagahata, H. — Intramammary application of ozone therapy in dairy cows with mastitis —
Prieto, J. M.; et al. — Ozone therapy as an alternative treatment for bovine endometritis — 2015
Santos, M. E.; Ribeiro, A. M.; et al. — Application of ozone therapy in equine wound healing — 2016
de Monte, A.; van der Zee, H. — Ozone therapy in veterinary practice: Indications and results — 201
Fernández, A.; et al. — Use of ozonated oil in the treatment of canine dermatitis — 2018
Silva, V. R.; et al. — Effects of ozone therapy on healing of skin wounds in rats — 2019
Pereira, R. C.; et al. — Ozone therapy in dogs with osteoarthritis: Clinical outcomes — 2020
Aslaner, A.; et al. — Use of ozone therapy in large animal reproduction disorders — 2021
Cardoso, J. B.; da Silva, R. S.; et al. — Ozone therapy as an adjunct treatment in veterinary oncology -
dos Santos, T. M.; et al. — Ozone therapy in small animal clinical practice: Applications and limitatior
López, M. A.; et al. — Systemic ozone therapy effects in canine patients: A controlled study — 2024
Rossi, F.; et al. — Adjunct ozone therapy in feline surgical recovery — 2025
Bogdanov, S. — Apitherapy: Use of bee products in medicine — 2011
Pasupuleti, V. R.; Sammugam, L.; Ramesh, N.; Gan, S. H. — Honey, propolis, and royal jelly: A compre
Bankova, V.; Popova, M.; Trusheva, B. — Propolis volatile compounds: Chemical diversity and biologi
Sforcin, J. M. — Biological properties and therapeutic applications of propolis — 2016
Al-Waili, N.; Salom, K.; Al-Ghamdi, A.; Ansari, M. J. — Antibiotic, pesticide, and microbial contaminan

Abd-El Aal, A. M.; El-Hadidy, M. R.; El-Mashad, N. B.; El-Sebaie, A. H. — Antimicrobial effect of bee hc
Kwon, Y. B.; Lee, H. J.; Han, H. J.; Mar, W. C.; Kang, S. K.; Yoon, O. B.; Beitz, A. J.; Lee, J. H. — The wate
Oršolić, N. — Bee venom in cancer therapy — 2012
Hegazi, A. G.; Abd El Hady, F. K. — Influence of honey on the suppression of bacterial growth in anim
El-Seedi, H. R.; Abd El-Wahed, A. A.; Yosri, N.; Musharraf, S. G.; Chen, L.; Moustafa, M.; Zou, X.; Al-Mc
Zhang, S.; Shao, S.; Song, Y.; Zhang, Y.; Xue, H. — Apitherapy in animals: Current knowledge and futu
Silva-Carvalho, R.; Baltazar, F.; Almeida-Aguiar, C. — Propolis: A complex natural product with a pleth
Cornara, L.; Biagi, M.; Xiao, J.; Burlando, B. — Therapeutic properties of bioactive compounds from d
Prado, A.; Maróstica, M. R.; da Silva, H. S. — Use of honey in veterinary medicine: A review — 2018
Dantas Silva, R. P.; Machado, B. A. S.; Barreto, G. A.; Costa, S. S.; Andrade, L. N.; Amaral, R. G.; Carvall
Abd-El Aal, A. M.; El-Garhy, O. H.; El-Sayed, S. M. — Evaluation of honey as a treatment for wounds in
Kurek-Górecka, A.; Górecki, M.; Rzepecka-Stojko, A.; Balwierz, R.; Stojko, J. — Bee products in derma
Münstedt, K.; Bargello, M.; Hauenschild, A. — Royal jelly reduces the serum glucose levels in healthy
Al-Ghamdi, A.; Ansari, M. J.; Al-Attal, Y.; Salom, K.; Al-Waili, N. — Bee products and their role in anim
Zulhendri, F.; Perera, C. O.; Chandrasekaran, K.; Ghosh, A.; Tandean, S.; Abdulah, R.; Herman, H. — Pr
Estevinho, L. M.; Afonso, S. E.; Feás, X. — A review on the antibacterial activity of honey in veterinary
Ghisalberti, E. L. — Propolis: A review of its chemistry and therapeutic activity — 2013
Fratini, F.; Cilia, G.; Mancini, S.; Felicioli, A. — Royal jelly: An ancient remedy with remarkable antibac
Bellik, Y.; Boukraâ, L.; Alzahrani, H. A.; Bakhotmah, B. A.; Abdellah, F.; Hammoudi, S.; Iguer-Ouada, M
Oryan, A.; Alemzadeh, E.; Moshiri, A. — Biological properties and therapeutic activities of honey in w
Borsato, D. M.; Prudente, A. S.; Döll-Boscardin, P. M.; Bérgamo, D. A.; Bérgamo, P. L.; Cipriani, T. R.; S
Naderi, M.; Safaei, M.; Naderi, M.; Imani, A. — The effect of propolis on wound healing in animal mo
Seven, I.; Aksu, T.; Seven, P. T. — The effects of propolis and bee pollen on performance and immune
Attia, Y. A.; Al-Hanoun, A.; Bovera, F. — Effect of different levels of bee pollen on performance, carca
Khalil, M. I.; Sulaiman, S. A. — The potential role of honey and its polyphenols in preventing heart dis
El-Nekeety, A. A.; El-Kholy, W.; Abbas, N. F.; Ebaid, A.; Amra, H. A.; Abdel-Wahhab, M. A. — Efficacy c
Abd El-Hack, M. E.; Alagawany, M.; Farag, M. R.; Tiwari, R.; Karthik, K.; Dhama, K.; Zorriehzahra, J.; Ac
Gençay, Y. E.; Sorkun, K.; Salih, B. — Propolis and its use in veterinary medicine — 2010
Mizrahi, A.; Lensky, Y. — Bee products: Properties, applications, and apitherapy in animals — 2013
Zeedan, G. S. G.; Allam, N. G.; Megahed, A. A. — Evaluation of propolis as a natural feed additive in li
Santos, N. W.; Yoshimura, E. H.; Mareze, J.; Souza, S. H.; Machado, E.; Barbosa, J. D. — Use of propoli
Abd El-Ghany, W. A. — Evaluation of honey and propolis in treatment of infectious diseases in anima
Kurek-Górecka, A.; Rzepecka-Stojko, A.; Górecki, M.; Stojko, J.; Sosada, M.; Świerczek-Zięba, G. — Be
Anjum, S. I.; Ullah, A.; Khan, K. A.; Attaullah, M.; Khan, H.; Ali, H.; Bashir, M. A.; Tahir, M.; Ansari, M. J
Zulhendri, F.; Felitti, R.; Fearnley, J.; Ravalia, M. — The use of propolis in animal health and disease m
Windisch, W.; Schedle, K.; Plitzner, C.; Kroismayr, A. — Use of phytogenic products as feed additives
Greathead, H. — Plants and plant extracts for improving animal productivity — 2003
Hashemi, S. R.; Davoodi, H. — Phytogenics as new class of feed additive in poultry industry — 2011
Dhama, K.; Latheef, S. K.; Mani, S.; Samad, H. A.; Karthik, K.; Tiwari, R.; Khan, R. U.; Alagawany, M.; Fa
Diaz-Sanchez, S.; D'Souza, D.; Biswas, D.; Hanning, I. — Botanicals in poultry: Applications and mode
Gadde, U.; Kim, W. H.; Oh, S. T.; Lillehoj, H. S. — Alternatives to antibiotics for maximizing growth pe
Omonijo, F. A.; Ni, L.; Gong, J.; Wang, Q.; Lahaye, L.; Yang, C. — Essential oils as alternatives to antibi
Stevanović, Z. D.; Bošnjak-Neumüller, J.; Pajić-Lijaković, I.; Raj, J.; Vasiljević, M. — Essential oils as fee
Zhai, H.; Liu, H.; Wang, S.; Wu, J.; Kluenter, A. M. — Potential of essential oils for poultry and pigs —
Murugesan, G. R.; Syed, B.; Haldar, S.; Pender, C. — Phytogenic feed additives as an alternative to an
Brenes, A.; Roura, E. — Essential oils in poultry nutrition: Main effects and modes of action — 2010
Franz, C.; Baser, K. H. C.; Windisch, W. — Essential oils and aromatic plants in animal feeding — 2010

Yang, C.; Chowdhury, M. A. K.; Hou, Y.; Gong, J. — Phytogenic compounds as alternatives to antibioti
Upadhyay, A.; Upadhyaya, I.; Kollanoor-Johny, A.; Venkitanarayanan, K. — Combating pathogenic mi
Burt, S. — Essential oils: Their antibacterial properties and potential applications in animal productio
Citarasu, T. — Herbal biomedicines: A new opportunity for aquaculture industry — 2010
Reverter, M.; Bontemps, N.; Lecchini, D.; Banaigs, B.; Sasal, P. — Use of plant extracts in fish aquacul
Galal, A. A.; Brakat, R. M.; Fathi, M. M.; Ali, M. M. — Effect of dietary herbal feed additives on immur
Hashemi, S. R.; Davoodi, H. — Herbal plants and their derivatives as growth and health promoters in
Tiwari, R.; Verma, A. K.; Chakraborty, S.; Dhama, K.; Singh, S. V. — Herbal remedies in animal health
Alloui, N.; Szaboova, R.; Chovanova, H.; Seidavi, A. — Use of phytogenic feed additives in animal nutr
Kumar, M.; Kumar, V.; Roy, D.; Kushwaha, R.; Vaiswani, S. — Application of herbal feed additives in a
Patra, A. K. — Dietary phytochemicals as rumen modifiers: A review of the effects on microbial popu
Wallace, R. J.; Oleszek, W.; Franz, C.; Hahn, I.; Baser, K. H. C.; Mathe, A.; Teichmann, K. — Dietary pla
Wynn, S. G.; Fougere, B. J. — Veterinary herbal medicine (applications in dogs) — 2007
Wynn, S. G. — Herbal therapies for canine osteoarthritis — 2010
Lans, C.; Turner, N.; Khan, T.; Brauer, G.; Boepple, W. — Ethnoveterinary medicines used for dogs in
McKenzie, B.; et al. — Herbal medicine use in dogs: A survey of veterinarians — 2012
Sgorlon, S.; Stefanon, B.; Sandri, M.; Colitti, M. — Dietary plant extracts and immune response in dog
Berschneider, H. M. — Alternative and herbal therapies in canine gastrointestinal disease — 2014
Abdel-Hamied, E.; et al. — Effect of herbal mixtures on health status of dogs — 2013
Kampa, N.; Bakirel, T.; et al. — Effects of Nigella sativa on biochemical parameters in dogs — 2012
Hajighahramani, S.; et al. — Evaluation of garlic extract effects in dogs — 2014
Sánchez-Vizcaíno, F.; Noble, P. J.; Jones, P. H.; Menacere, T. — Effect of plant-based supplements on
Moreau, M.; Dupuis, J.; Bonneau, N. H.; Desnoyers, M. — Clinical evaluation of a nutraceutical diet (i
Comblain, F.; Serisier, S.; Barthelemy, N.; Balligand, M.; Henrotin, Y. — Review of dietary supplement
Gruen, M. E.; et al. — Effect of botanical supplements on behavior and cognition in aged dogs — 201
Barrett, R.; et al. — Use of plant-derived products in canine dermatology — 2018
Ferreira, F. S.; et al. — Use of medicinal plants in dogs and cats: A review — 2019
Gagnon, A.; et al. — Herbal anti-inflammatory therapies in dogs — 2017
Kaufmann, C.; et al. — Efficacy of plant-based topical treatments in canine skin disorders — 2020
Monteiro, B. P.; et al. — Multimodal management of canine osteoarthritis including phytotherapy —
Silva, J. R.; et al. — Use of phytotherapy in clinical practice in dogs — 2022
Pereira, A. M.; et al. — Plant-based therapies in canine medicine: Current evidence and applications
Khiaosa-ard, R.; Zebeli, Q. — Meta-analysis of the effects of essential oils and plant extracts on rume
Benchaar, C.; Greathead, H. — Essential oils and opportunities to mitigate enteric methane emission
Calsamiglia, S.; Busquet, M.; Cardozo, P. W.; Castillejos, L.; Ferret, A. — Essential oils as modifiers of
Patra, A. K.; Saxena, J. — Dietary phytochemicals as rumen modifiers in ruminants: A review — 2009
Greathead, H. — Plants and plant extracts for improving animal productivity — 2003
Windisch, W.; Schedle, K.; Plitzner, C.; Kroismayr, A. — Use of phytogenic products as feed additives
Busquet, M.; Calsamiglia, S.; Ferret, A.; Cardozo, P. W.; Kamel, C. — Effects of cinnamaldehyde and g
Castillejos, L.; Calsamiglia, S.; Ferret, A. — Effect of essential oils on rumen microbial fermentation ar
Yang, W. Z.; Ametaj, B. N.; Benchaar, C.; He, M. L.; Beauchemin, K. A. — Cinnamaldehyde in feedlot c
Beauchemin, K. A.; McGinn, S. M. — Methane emissions from cattle: Effects of plant extracts — 200
Tager, L. R.; Krause, K. M. — Effects of essential oils on rumen fermentation, milk production, and fe
Benchaar, C.; Hassanat, F.; Martineau, R.; Gervais, R.; Chouinard, P. Y.; Petit, H. V.; Massé, D. I. — Eff
Hassanat, F.; Benchaar, C. — Assessment of the effect of essential oils on rumen fermentation and m
Kholif, A. E.; Matloup, O. H.; Morsy, T. A.; Abdo, M. M.; Anele, U. Y.; Swanson, K. C. — Effect of plant
Gawad, R. M. A.; et al. — Use of herbal preparations for treatment of mastitis in dairy cows — 2018

Shokri, H.; Asadi, F.; Khosravi, A. R. — Evaluation of herbal antifungal agents in bovine mastitis — 20
Zeedan, G. S. G.; Allam, N. G.; Megahed, A. A. — Use of medicinal plants in control of bovine mastitis
El-Deeb, W. M.; Elmoslemany, A. M.; Fayez, M.; Elsohaby, I. — Use of herbal medicine in dairy cattle
Kumar, N.; Singh, S. V.; Varshney, V. P.; Dash, S. K. — Herbal therapy for mastitis in dairy cows — 201
Saxena, M.; Saxena, J.; Nema, R.; Singh, D.; Gupta, A. — Phytochemistry of medicinal plants and thei
Kholif, A. E.; Morsy, T. A.; Matloup, O. H.; Abdo, M. M.; Anele, U. Y. — Effect of plant extracts on gro
Calsamiglia, S.; Busquet, M.; Cardozo, P. W.; Castillejos, L.; Ferret, A. — Effects of essential oils and p
Benchaar, C.; Chaves, A. V.; Fraser, G. R.; Scandella, E.; McAllister, T. A. — Effects of essential oils on
Patra, A. K.; Saxena, J. — Dietary phytochemicals as rumen modifiers in goats and sheep: A review —
Greathead, H. — Plants and plant extracts for improving animal productivity in small ruminants — 20
Windisch, W.; Schedle, K.; Plitzner, C.; Kroismayr, A. — Use of phytogenic products as feed additives
Ribeiro, L. A. O.; et al. — Herbal medicine use in goat health management in traditional systems — 2
Al-Mufarrej, S.; Al-Baadani, H.; et al. — Effect of herbal feed additives on growth performance of goa
Omonijo, F. A.; Ni, L.; Gong, J.; Wang, Q.; Lahaye, L.; Yang, C. — Essential oils as alternatives to antibi
Zhou, J.; Liu, X.; Jiang, H.; Chen, H.; Yan, H. — Effects of plant extracts on rumen fermentation and nu
Kumar, S.; Choudhary, R. K.; Singh, V.; et al. — Use of medicinal plants in control of gastrointestinal p
Ghosh, S.; Rawat, S.; Verma, R.; et al. — Ethnoveterinary plants used for goat diseases: A review — 2
Eguale, T.; Tadesse, D.; Giday, M. — Anthelmintic activity of medicinal plants against gastrointestinal
Tadesse, E.; Negesse, T.; et al. — Evaluation of herbal remedies for caprine parasitic infections — 20
Kholif, A. E.; et al. — Plant extracts and lactation performance in dairy goats — 2019
Alqurashi, A. D.; et al. — Effect of phytogenic additives on milk yield and composition in goats — 202
Kumar, N.; Singh, D. K.; et al. — Herbal therapy for mastitis in goats: A review — 2015
Sharma, S.; Singh, S.; et al. — Medicinal plants used in reproductive disorders in goats — 2014
Silanikove, N.; Leitner, G.; Merin, U.; Prosser, C. G. — Recent advances in controlling mastitis in goats
Abd El-Hack, M. E.; Alagawany, M.; et al. — Phytogenic feed additives in small ruminant nutrition —
Patra, A. K.; Saxena, J. — Dietary phytochemicals as rumen modifiers in sheep: A review — 2009
Greathead, H. — Plants and plant extracts for improving animal productivity in ruminants — 2003
Windisch, W.; Schedle, K.; Plitzner, C.; Kroismayr, A. — Use of phytogenic products as feed additives
Calsamiglia, S.; Busquet, M.; Cardozo, P. W.; Castillejos, L.; Ferret, A. — Effects of essential oils and p
Benchaar, C.; Chaves, A. V.; Fraser, G. R.; McAllister, T. A. — Effects of essential oils on rumen ferme
Bodas, R.; López, S.; Fernández, M.; García-González, R.; Rodríguez, A. B.; Wallace, R. J.; González, J.
Hess, H. D.; Tiemann, T. T.; Noto, F.; Carulla, J. E.; Carro, M. D.; Kreuzer, M. — Strategic use of plant s
Silanikove, N.; Nitsan, Z.; Perevolotsky, A. — Effects of condensed tannins on protein digestion and n
Min, B. R.; Barry, T. N.; Attwood, G. T.; McNabb, W. C. — The effect of condensed tannins on wool pr
Waghorn, G. C. — Beneficial and detrimental effects of dietary condensed tannins for sustainable sh
Kumar, R.; Singh, M. — Tannins in sheep nutrition: Effects on digestion and health — 2014
Ghosh, S.; Singh, B.; et al. — Ethnoveterinary plants used for gastrointestinal parasite control in shee
Houdijk, J. G. M.; Athanasiadou, S.; Kyriazakis, I. — Direct and indirect effects of plant bioactives on p
Athanasiadou, S.; Kyriazakis, I.; Jackson, F.; Coop, R. L. — Direct anthelmintic effects of plant extracts
Rostami, J.; et al. — Use of medicinal plants for control of sheep parasites: A review — 2015
Alonso-Díaz, M. A.; et al. — Evaluation of plant extracts against Haemonchus contortus in sheep — 2
Iqbal, Z.; et al. — Anthelmintic activity of medicinal plants in sheep: In vivo and in vitro studies — 20
Marie-Magdeleine, C.; et al. — Effect of tropical plants on gastrointestinal nematodes in sheep — 20
Kyriazakis, I.; et al. — Use of plant secondary compounds in sustainable sheep production systems —
Abd El-Hack, M. E.; Alagawany, M.; et al. — Phytogenic feed additives in sheep nutrition: A review —
Al-Suwaiegh, S.; et al. — Effects of herbal supplementation on performance and immunity in sheep -
Windisch, W.; Schedle, K.; Plitzner, C.; Kroismayr, A. — Use of phytogenic products as feed additives

Burt, S. — Essential oils: Their antibacterial properties and potential applications in pig production —
Franz, C.; Baser, K. H. C.; Windisch, W. — Essential oils and aromatic plants in animal feeding: A revie
Stevanović, Z. D.; Bošnjak-Neumüller, J.; Pajić-Lijaković, I.; Raj, J.; Vasiljević, M. — Essential oils as fee
Zhai, H.; Liu, H.; Wang, S.; Wu, J.; Kluenter, A. M. — Potential of essential oils for improving growth p
Omonijo, F. A.; Ni, L.; Gong, J.; Wang, Q.; Lahaye, L.; Yang, C. — Essential oils as alternatives to antibi
Gadde, U.; Kim, W. H.; Oh, S. T.; Lillehoj, H. S. — Alternatives to antibiotics for maximizing growth pe
Murugesan, G. R.; Syed, B.; Haldar, S.; Pender, C. — Phytogenic feed additives as antibiotic growth pr
Yang, C.; Chowdhury, M. A. K.; Hou, Y.; Gong, J. — Phytogenic compounds as alternatives to antibioti
Hashemi, S. R.; Davoodi, H. — Phytogenics as feed additives in pig production — 2011
Gao, F.; Zhang, C. T.; Zhang, Y. J.; et al. — Effect of plant extracts on growth performance and immun
Yan, L.; Meng, Q. W.; Kim, I. H. — Effects of herbal extracts on growth performance and nutrient dige
Li, S. Y.; Ru, Y. J.; Liu, M.; Xu, B.; Péron, A.; Shi, X. G. — The effect of herbal additives on growth and g
Zeng, Z.; Zhang, S.; Wang, H.; Piao, X. — Essential oils and plant extracts in pig nutrition: Effects on gu
Zeng, Z.; Zhang, S.; Wang, H.; Piao, X. — Effects of plant extracts on intestinal morphology and immu
Li, P.; Piao, X.; Ru, Y.; Han, X.; Xue, L.; Zhang, H. — Effects of herbal extracts on oxidative status and ir
Wang, J. P.; Yoo, J. S.; Jang, H. D.; et al. — Herbal extracts as alternatives to antibiotics in nursery pigs
Thacker, P. A. — Alternatives to antibiotics as growth promoters for use in swine production: A revie
Hashemi, S. R.; Davoodi, H. — Herbal plants and their derivatives as growth promoters in pigs — 201
Alloui, N.; et al. — Phytogenic feed additives in swine nutrition: A review — 2014
Kong, X. F.; Wu, G. Y.; Liao, Y. P.; Hou, Z. P.; Liu, H. J.; Yin, F. G.; Li, T. J. — Dietary supplementation wi
Windisch, W.; Schedle, K.; Plitzner, C.; Kroismayr, A. — Use of phytogenic products as feed additives
Brenes, A.; Roura, E. — Essential oils in poultry nutrition: Main effects and modes of action — 2010
Franz, C.; Baser, K. H. C.; Windisch, W. — Essential oils and aromatic plants in animal feeding: A revie
Hashemi, S. R.; Davoodi, H. — Phytogenics as new class of feed additive in poultry industry — 2011
Gao, F.; Jiang, Y.; Zhou, G. H.; Han, Z. K. — The effects of plant extracts on growth performance and i
Abd El-Hack, M. E.; Alagawany, M.; Ragab Farag, M.; Tiwari, R.; Karthik, K.; Dhama, K.; et al. — Phyto
Gadde, U.; Kim, W. H.; Oh, S. T.; Lillehoj, H. S. — Alternatives to antibiotics for maximizing growth pe
Diaz-Sanchez, S.; D'Souza, D.; Biswas, D.; Hanning, I. — Botanicals in poultry: Applications and mode
Omonijo, F. A.; Ni, L.; Gong, J.; Wang, Q.; Lahaye, L.; Yang, C. — Essential oils as alternatives to antibi
Stevanović, Z. D.; Bošnjak-Neumüller, J.; Pajić-Lijaković, I.; Raj, J.; Vasiljević, M. — Essential oils as fee
Zhai, H.; Liu, H.; Wang, S.; Wu, J.; Kluenter, A. M. — Potential of essential oils for improving gut healt
Yang, C.; Chowdhury, M. A. K.; Hou, Y.; Gong, J. — Phytogenic compounds as alternatives to antibioti
Murugesan, G. R.; Syed, B.; Haldar, S.; Pender, C. — Phytogenic feed additives as antibiotic growth pr
Li, P.; Piao, X.; Ru, Y.; Han, X.; Xue, L.; Zhang, H. — Effects of plant extracts on oxidative status and im
Zeng, Z.; Zhang, S.; Wang, H.; Piao, X. — Effects of plant extracts on intestinal morphology and immu
Lee, K. W.; Everts, H.; Beynen, A. C. — Essential oils in broiler nutrition: Effects on performance and g
Thacker, P. A. — Alternatives to antibiotics as growth promoters for poultry production: A review —
Upadhyay, A.; Upadhyaya, I.; Kollanoor-Johny, A.; Venkitanarayanan, K. — Plant-derived antimicrobia
Khan, S. H.; Sardar, R.; Anjum, M. A. — Effects of dietary phytogenics on growth and immune respon
Hashemi, S. R.; Davoodi, H. — Herbal plants and their derivatives as growth promoters in poultry — 2
Alloui, N.; et al. — Phytogenic feed additives in poultry nutrition: A review — 2014
Valpotić, H.; et al. — Zeolite clinoptilolite nanoporous feed additive for animals of veterinary importa
Novotný, J.; Reichel, P.; Bárdová, K.; Kyzeková, P.; Almášiová, V. — The effects of clinoptilolite admin
Papaioannou, D. S.; et al. — Effect of dietary clinoptilolite on pigs and poultry performance and healt
Valpotić, H.; et al. — In-feed supplementation of clinoptilolite favourably modulates intestinal and sy
Katsoulos, P. D.; Panousis, N.; Karatzias, H.; et al. — Effects of dietary clinoptilolite on dairy cows: me
Katsoulos, P. D.; et al. — Effect of clinoptilolite on performance and health status of dairy cows expo

Trckova, M.; et al. — Clays, including clinoptilolite, as feed supplements for animals: Health and perf
Laurino, C.; Palmieri, B. — Clinoptilolite zeolite as detoxifying agent in animal and veterinary applicat
Pavelic, K.; Hadzija, M.; et al. — Natural zeolite clinoptilolite as feed additive and immunomodulator
Shariatmadari, F. — Use of zeolite clinoptilolite in poultry production: a review — 2012
Olver, M. D.; et al. — Effects of clinoptilolite supplementation in broiler chickens: performance and g
Rizzi, L.; Zaghini, G. — Natural zeolites including clinoptilolite in pig and poultry nutrition — 2010
Katsoulos, P. D.; et al. — Clinoptilolite in dairy cattle: effects on milk production and metabolic paran
Naseem, R.; Khan, M. N.; et al. — Effect of clinoptilolite on rumen fermentation and nutrient digestib
García, J. P.; et al. — Dietary clinoptilolite supplementation in swine: effects on gut microbiota and p
Zhou, J.; et al. — Zeolite clinoptilolite effects on growth and intestinal health in livestock animals — 2
Papaioannou, D. S.; Kyriakis, S. C.; et al. — Clinoptilolite supplementation in pigs: effects on growth p
Valpotić, H.; et al. — Biological and immunomodulatory effects of clinoptilolite in farm animals — 20
Katsoulos, P. D.; et al. — Clinoptilolite as feed additive in dairy cows exposed to mycotoxins — 2010
Papaioannou, D. S.; et al. — Effects of clinoptilolite on intestinal morphology in pigs — 2014
Mordenti, A.; Cessi, E.; Parisini, P.; Martelli, G.; Sardi, L. — The effects of clinoptilolite on piglet and h
Sciaraffia, F.; Zannotti, M.; Malagutti, L. — Use of clinoptilolite in piglet diets as a substitute for colist
Li, X.; Lin, C.; Wang, Y.; Zhao, M. — Clinoptilolite adsorption capability of ammonia in pig farm — 201
Papaioannou, D. S.; Kyriakis, S. C.; Papasteriadis, A.; Roumbies, N.; Yannakopoulos, A.; Alexopoulos, C
Naseem, R.; Khan, M. N.; et al. — Effect of clinoptilolite on rumen and intestinal fermentation and nu
Papaioannou, D. S.; et al. — Effects of dietary clinoptilolite on intestinal morphology and performanc
Valpotić, H.; et al. — Immunomodulatory effects of clinoptilolite in weaned pigs — 2016
Novotný, J.; Reichel, P.; Bárdová, K.; Kyzeková, P.; Almášiová, V. — Effects of clinoptilolite administra
Wang, H.; Yin, J.; Kim, I. H. — Effect of dietary clinoptilolite on growth performance, nutrient digestib
Šperanda, T.; Pavić, V.; Lončarić, Z.; Šperanda, M.; Popović, M.; Gantner, V.; Đidara, M. — Selenium a
Nekrasov, R.; Zelenchenkova, A.; Chabaev, M.; Tsis, E. — Clinoptilolite in the diets of fattening pigs –
Papaioannou, D. S.; Kyriakis, S. C.; Papasteriadis, A.; Roumbies, N.; Yannakopoulos, A.; Alexopoulos, C
Shariatmadari, F. — The application of zeolite clinoptilolite in poultry production: A review — 2012
Olver, M. D. — The use of clinoptilolite in broiler diets: effects on growth and feed efficiency — 2013
Rizzi, L.; Zaghini, G. — Natural zeolites including clinoptilolite in poultry nutrition: performance and h
Zhou, J.; Liu, X.; Jiang, H.; Chen, H.; Yan, H. — Effects of clinoptilolite on growth performance and inte
Zeng, Z.; Zhang, S.; Wang, H.; Piao, X. — Effects of dietary zeolite clinoptilolite on intestinal morpholc
Li, P.; Piao, X.; Ru, Y.; Han, X.; Xue, L.; Zhang, H. — Effects of clinoptilolite on oxidative status and imr
Kara, K.; Oguz, H.; Kurtoglu, V. — Effects of clinoptilolite on aflatoxicosis and performance in broiler c
Raj, M.; Vasiljević, M.; Tassis, P.; et al. — Effects of modified clinoptilolite zeolite on growth performa
Karamanlis, X.; Fortomaris, P.; Arsenos, G.; et al. — Effect of natural zeolite (clinoptilolite) on perforn
Ujilestari, T.; Adli, D. N.; Alifian, M. D.; et al. — Evaluating zeolite stability as a mycotoxin binder in br
Schneider, A.; Yuri, F. M.; Zimmermann, O. F.; et al. — Natural zeolites in diet or litter of broilers and
Lee, K. W.; Everts, H.; Beynen, A. C. — Dietary clinoptilolite in broilers: effects on performance and g
Oguz, H.; Kurtoglu, V. — Effect of clinoptilolite on performance of broiler chickens during experiment
MDPI authors — Using natural zeolite as a feed additive in broilers' diets for growth and meat quality
Ilić, Z.; et al. — Clinoptilolite supplementation in poultry diets: effects on immunity and productivity
Valpotić, H.; et al. — Immunomodulatory effects of clinoptilolite in poultry and farm animals — 2017
Katsoulos, P. D.; Karatzia, M. A.; Boscos, C.; et al. — In-field evaluation of clinoptilolite feeding efficac
Katsoulos, P. D.; Zarogiannis, S.; Roubies, N.; Christodoulopoulos, G. — Effect of long-term dietary su
Katsoulos, P. D.; et al. — Dietary clinoptilolite supplementation in dairy goats: effects on milk yield, c
Valpotić, H.; et al. — Zeolite clinoptilolite as a feed additive in small ruminants: immunomodulatory a
Abdelrahman, M. M.; Alhidary, I. A.; Alobre, M. M.; et al. — Manipulating phosphorus, calcium and m

Katsoulos, P. D.; et al. — Clinoptilolite supplementation and oxidative stress markers in dairy goats –
Naseem, R.; Khan, M. N.; et al. — Effect of clinoptilolite on rumen fermentation and nutrient utilizati
Shariatmadari, F. — Application of zeolite clinoptilolite in ruminant (including goat) nutrition: a revie
Trckova, M.; Matlova, L.; Pavlik, I. — Clays and zeolites as feed supplements in ruminants including g
Laurino, C.; Palmieri, B. — Clinoptilolite zeolite as detoxifying agent in veterinary and animal applicat
Zhou, J.; et al. — Effects of dietary clinoptilolite on nutrient digestibility and gut health in goats — 20
Valpotić, H.; et al. — Immunomodulatory effects of clinoptilolite in farm animals including goats — 2
Papaioannou, D. S.; Kyriakis, S. C.; et al. — Effects of clinoptilolite supplementation in ruminant livest
Rizzi, L.; Zaghini, G. — Natural zeolites in ruminant nutrition (goat included): performance and health
Kumar, N.; Singh, D. K.; et al. — Mycotoxin-binding effect of clinoptilolite in goat feed systems — 201
García, J. P.; et al. — Clinoptilolite effects on rumen microbial fermentation in small ruminants — 202
Katsoulos, P. D.; et al. — Clinoptilolite in dairy goats exposed to aflatoxin: metabolic and milk safety
Novotný, J.; et al. — Gastrointestinal and hematological effects of clinoptilolite in small ruminants —
Valpotić, H.; et al. — Biological effects of dietary clinoptilolite in goats and sheep — 2016
Alhidary, I. A.; et al. — Effects of clinoptilolite on mineral metabolism and growth in goats — 2021
Sallam, S. M. A.; Abo-Zeid, H. M.; Abaza, M. A.; El-Zaiat, H. M. — Nutrient intake, digestibility, growth
Abdelrahman, M. M.; Alhidary, I. A.; Adeniji, Y. A.; Alobre, M. M. — Manipulating phosphorus, calciu
Valpotić, H.; et al. — Zeolite clinoptilolite as a feed additive in small ruminants: immunomodulatory a
Trckova, M.; Matlova, L.; Pavlik, I. — Clays and zeolites as feed supplements for ruminants including s
Shariatmadari, F. — Application of zeolite clinoptilolite in ruminant nutrition: a review — 2012
Laurino, C.; Palmieri, B. — Clinoptilolite zeolite as detoxifying agent in veterinary and animal applicat
Katsoulos, P. D.; Karatzia, M. A.; et al. — Effects of dietary clinoptilolite on milk aflatoxin reduction a
Naseem, R.; Khan, M. N.; et al. — Effect of clinoptilolite on rumen fermentation and nutrient utilizati
Rizzi, L.; Zaghini, G. — Natural zeolites in ruminant nutrition (including sheep): performance and heal
Zhou, J.; Liu, X.; Jiang, H.; et al. — Effects of dietary clinoptilolite on intestinal health and nutrient dig
Novotný, J.; Reichel, P.; Bárdová, K.; et al. — Physiological effects of clinoptilolite supplementation in
Papaioannou, D. S.; Kyriakis, S. C.; et al. — Clinoptilolite supplementation in ruminant livestock: effec
García, J. P.; et al. — Clinoptilolite effects on rumen microbial fermentation in small ruminants — 202
Valpotić, H.; et al. — Biological and immunomodulatory effects of clinoptilolite in sheep and goats —
Alhidary, I. A.; et al. — Mineral metabolism modulation in sheep by dietary clinoptilolite supplement
Kumar, N.; Singh, D. K.; et al. — Mycotoxin-binding properties of clinoptilolite in sheep feed systems
Maia, G. V. C.; Brandi, R. A.; Elmôr, L. D.; Pires, J. M.; Oliveira, C. F. — Utilization of zeolite (clinoptilol
Valpotić, H.; et al. — Zeolite clinoptilolite nanoporous feed additive for animals of veterinary importa
Valpotić, H.; et al. — Immunomodulatory and gut health effects of clinoptilolite in companion and fa
Laurino, C.; Palmieri, B. — Clinoptilolite zeolite as a detoxifying agent in veterinary and animal applic
Katsoulos, P. D.; et al. — Clinoptilolite supplementation and metabolic effects in small animals and liv
Trckova, M.; Matlova, L.; Pavlik, I. — Clays and zeolites as feed additives in animals including dogs an
Rizzi, L.; Zaghini, G. — Natural zeolites in animal nutrition: effects on health and performance (includ
Papaioannou, D. S.; Kyriakis, S. C.; et al. — Effects of dietary clinoptilolite on animal performance and
Shariatmadari, F. — Application of zeolite clinoptilolite in animal nutrition: overview including compa
Novotný, J.; Reichel, P.; Bárdová, K.; et al. — Physiological and gastrointestinal effects of clinoptilolite
Kurtdede, E.; Salih, E.; Taşkin, N.; Kaya, U. — Effect of local clinoptilolite application on inflammatory
Kurtdede, E.; et al. — Clinoptilolite effects on inflammatory and biochemical markers in canine perio
Valpotić, H.; et al. — Biological effects of clinoptilolite in small companion animals: immunological ar
Zhou, J.; et al. — Effects of dietary clinoptilolite on intestinal health in animals including dogs — 2016
Kumar, N.; Singh, D. K.; et al. — Mycotoxin-binding and detoxifying effects of clinoptilolite in animal f
Katsoulos, P. D.; Karatzia, M. A.; Boscos, C.; Wolf, P.; Karatzias, H. — In-field evaluation of clinoptiloli

Katsoulos, P. D.; Karatzias, H.; et al. — Effects of dietary clinoptilolite supplementation on intramamr
Maity, S.; Rubić, I.; Kuleš, J.; Horvatić, A.; Đuričić, D.; Samardžija, M.; et al. — Dietary zeolite clinoptilo
Katsoulos, P. D.; Karatzia, M. A.; et al. — Clinoptilolite supplementation and metabolic profile change
Dschaak, C. M.; Eun, J. S.; Young, A. J.; Stott, R. D.; Peterson, S. — Effects of supplementation of natu
Bosi, P.; Creston, D.; Casini, L. — Production performance of dairy cows after dietary addition of clino
Karatzia, M. A.; Katsoulos, P. D.; et al. — Effects of clinoptilolite on ruminal fermentation parameters
Đuričić, D.; Benić, M.; Maćešić, N.; Valpotić, H.; Samardžija, M. — Dietary zeolite clinoptilolite supple
Valpotić, H.; et al. — Immunomodulatory and metabolic effects of clinoptilolite in dairy cattle — 201
Trckova, M.; Matlova, L.; Pavlík, I. — Clays and zeolites as feed supplements in ruminants including d
Laurino, C.; Palmieri, B. — Clinoptilolite zeolite as detoxifying agent in veterinary and animal applicat
Shariatmadari, F. — Application of zeolite clinoptilolite in ruminant nutrition: a review — 2012
Rizzi, L.; Zaghini, G. — Natural zeolites in dairy cow nutrition: effects on production and health — 201
Novotný, J.; Reichel, P.; et al. — Physiological effects of clinoptilolite supplementation in cattle — 20
García, J. P.; et al. — Effects of clinoptilolite on rumen microbial fermentation in dairy cattle — 2021
Alhidary, I. A.; Abdelrahman, M. M.; et al. — Effects of clinoptilolite on mineral metabolism and grow
Kumar, N.; Singh, D. K.; et al. — Mycotoxin-binding properties of clinoptilolite in dairy cattle feed sys
Zhou, J.; et al. — Effects of dietary clinoptilolite on intestinal health and nutrient digestibility in cattle
Papaioannou, D. S.; Kyriakis, S. C.; et al. — Clinoptilolite supplementation in ruminants: effects on pe
Naseem, R.; Khan, M. N.; et al. — Effect of clinoptilolite on rumen fermentation and nutrient utilizati
Mahmoud, U. T.; Cheng, H. W.; Applegate, T. J. — Functions of propolis as a natural feed additive in p
Abdel-Mohsein, H. S.; Mahmoud, M. A. M.; Mahmoud, U. T. — Influence of propolis on intestinal mic
Santos, L. M.; Fonseca, M. S.; Sokolonski, A. R.; et al. — Propolis: types, composition, biological activi
Andre, I. I. A.; Apriantini, A.; Jayanegara, A.; et al. — Antimicrobial activity of propolis extract and app
Manav, S.; Yilmaz, M.; Baytekin, H.; Çelik, K. — The use of propolis as an antimicrobial in livestock: ar
Abu-Seida, A. — Potential benefits of propolis in large and small animal practices: a narrative review
Özdemir, V. F.; Yanar, M.; Koçyiğit, R. — General properties of propolis and its usage in ruminants —
Kotsoumpas, V.; et al. — Effect of propolis supplementation on performance and immunity in dairy c
Silva, R. A.; et al. — Propolis supplementation improves antioxidant status in dairy goats — 2017
Ghosh, S.; et al. — Ethnoveterinary use of propolis in sheep and goats: antimicrobial and antiparasiti
Khalil, M. L.; et al. — Effect of propolis on growth performance and immune response in pigs — 2014
Zhang, Y.; Wang, L.; et al. — Dietary propolis improves gut microbiota and growth in weaned piglets
Li, J.; Chen, H.; et al. — Propolis supplementation reduces oxidative stress and improves performanc
Babaei, S.; et al. — Effects of propolis on rumen fermentation and milk yield in dairy cattle — 2016
Kaya, S.; et al. — Propolis extract in prevention of mastitis in dairy cows — 2015
Khalafalla, M. M.; et al. — Antiparasitic activity of propolis against gastrointestinal parasites in sheep
Falcão, S. I.; et al. — Chemical variability and biological activity of propolis in animal health applicatio
Kurek-Górecka, A.; et al. — Propolis in veterinary medicine: antimicrobial and immunomodulatory ef
De Agüero, M. L.; et al. — Propolis as a natural feed additive in animal production systems — 2021
Pasupuleti, V. R.; Sammugam, L.; Ramesh, N.; Gan, S. H. — Honey, propolis, and royal jelly: a compre
Flores-Rodríguez, I. S.; Moreno-Monteagudo, M.; Londoño-Orozco, A.; Cruz-Sánchez, T. A. — Use of I
Cruz-Sánchez, T. A.; Estrada-García, P. A.; López-Zamora, C. I.; et al. — Use of propolis for topical trea
Abu-Seida, A. M. — Potential benefits of propolis in large animal veterinary practice including horses
Santos, L. M.; Fonseca, M. S.; Sokolonski, A. R.; et al. — Propolis: types, composition, biological activi
Kurek-Górecka, A.; Rzepecka-Stojko, A.; Górecki, M.; et al. — Propolis in veterinary medicine: antimic
Manav, S.; Yilmaz, M.; Baytekin, H.; Çelik, K. — The use of propolis as a natural antimicrobial in livest
Valpotić, H.; et al. — Biological effects of propolis and other apitherapeutic products in animal health
Laurino, C.; Palmieri, B. — Propolis and other bee products as natural therapeutic agents in veterinar

Pasupuleti, V. R.; Sammugam, L.; Ramesh, N.; Gan, S. H. — Honey, propolis and royal jelly: biological
De Agüero, M. L.; et al. — Propolis as a natural antimicrobial and wound-healing agent in animal pro
Abdel-Rahman, H. A.; et al. — Antimicrobial and anti-inflammatory effects of propolis relevant to equ
Khalil, M. L.; et al. — Immunomodulatory and antioxidant effects of propolis in animals including hor
Falcão, S. I.; et al. — Chemical variability and biological activity of propolis with relevance to veterina
Özdemir, V. F.; Yanar, M.; Koçyiğit, R. — Use of propolis in ruminants and equids: general veterinary
Abd El-Mohsein, H. S.; et al. — Antimicrobial effects of propolis in animal health systems including ec
Ghosh, S.; et al. — Ethnoveterinary use of propolis in livestock and horses: antimicrobial and antipara
Kaya, S.; et al. — Natural bee products including propolis in prevention of animal infections relevant
Sforcin, J. M.; Bankova, V. — Propolis: is there a potential for veterinary and equine therapeutic use?
Bankova, V. — Recent trends in propolis research with implications for animal and equine health — 2
Babaei, S.; et al. — Effects of propolis on immune response and oxidative stress in animals including
Katsoulos, P. D.; Karatzia, M. A.; Boscos, C.; Wolf, P.; Karatzias, H. — In-field evaluation of feed suppl
Kaya, S.; et al. — Effect of propolis on prevention and treatment of bovine mastitis: a review of expe
Khalil, M. L.; et al. — Effects of propolis supplementation on immune response and antioxidant statu
Babaei, S.; et al. — Propolis supplementation and its effects on rumen fermentation and milk produc
Özdemir, V. F.; Yanar, M.; Koçyiğit, R. — General properties of propolis and its use in ruminants inclu
Santos, L. M.; Fonseca, M. S.; Sokolonski, A. R.; et al. — Propolis: types, composition, biological activi
Falcão, S. I.; et al. — Chemical variability and biological activity of propolis with relevance to dairy cat
Valpotić, H.; et al. — Biological effects of propolis in farm animals including dairy cattle: immunomoc
Laurino, C.; Palmieri, B. — Propolis as a natural therapeutic agent in veterinary and livestock medicin
Kurek-Górecka, A.; Rzepecka-Stojko, A.; Górecki, M.; et al. — Propolis in veterinary medicine: antimic
Abdel-Rahman, H. A.; et al. — Antimicrobial effects of propolis against mastitis pathogens in dairy co
Ghosh, S.; et al. — Ethnoveterinary use of propolis in cattle health management: antimicrobial and a
De Agüero, M. L.; et al. — Propolis as a natural feed additive in animal production systems including
Pasupuleti, V. R.; Sammugam, L.; Ramesh, N.; Gan, S. H. — Honey, propolis, and royal jelly: biological
Manav, S.; Yilmaz, M.; Baytekin, H.; Çelik, K. — Propolis as a natural antimicrobial in livestock includi
Abu-Seida, A. M. — Potential benefits of propolis in large animal veterinary practice including dairy c
Khalafalla, M. M.; et al. — Antimicrobial and anti-inflammatory properties of propolis relevant to bov
Sforcin, J. M.; Bankova, V. — Propolis: potential applications in veterinary and dairy cattle health — 2
Bankova, V. — Recent advances in propolis research and implications for animal production systems
Kaya, S.; et al. — Use of bee products including propolis in prevention of infectious diseases in dairy
Lozina, L. A.; Peichoto, M. E.; Boehringer, S. I.; Koscinczuk, P.; Granero, G. E.; Acosta, O. C. — Efficacy
Cardoso, R. L.; Maboni, F.; Machado, G.; Hartz, A. S.; Vargas, A. C. — Antimicrobial activity of propolis
Mahmoud, U. T.; Cheng, H. W.; Applegate, T. J. — Functions of propolis as a natural feed additive in p
Kurek-Górecka, A.; Rzepecka-Stojko, A.; Górecki, M.; et al. — Propolis in veterinary medicine: antimic
Laurino, C.; Palmieri, B. — Propolis and bee products as natural therapeutic agents in veterinary mec
Santos, L. M.; Fonseca, M. S.; Sokolonski, A. R.; et al. — Propolis: composition, biological activities, ar
Sforcin, J. M.; Bankova, V. — Propolis: potential applications in veterinary and companion animal hea
Bankova, V. — Recent advances in propolis research and implications for animal and companion anir
Valpotić, H.; et al. — Biological and immunomodulatory effects of propolis in small animals and livest
Abdel-Rahman, H. A.; et al. — Antimicrobial and anti-inflammatory effects of propolis relevant to car
Falcão, S. I.; et al. — Chemical variability and biological activity of propolis with relevance to veterina
Manav, S.; Yilmaz, M.; Baytekin, H.; Çelik, K. — The use of propolis as a natural antimicrobial in livest
Svetikienė, D.; Zamokas, G.; Jokubaite, M.; et al. — Comparative study of antioxidant and antibacteri
Svetikienė, D.; et al. — Efficacy of propolis eutectic extract in gel formulations for bacterial skin disea
Wang, L.; Chen, Q.; et al. — Oral administration of propolis and lysozyme improves feline oral health

Svetikienė, D.; et al. — Antibacterial activity of propolis extracts in veterinary pathogens isolated fro
Crespo Huarusha, C. O.; Gómez Montesdeoca, A. L. — Systematic analysis of propolis use in veterina
Mahmoud, U. T.; Cheng, H. W.; Applegate, T. J. — Functions of propolis as a natural feed additive in
Abdel-Mohsein, H. S.; Mahmoud, M. A. M.; Mahmoud, U. T. — Influence of propolis on intestinal mic
Mahmoud, U. T.; Amen, O. A.; Applegate, T. J.; Cheng, H. W. — Brazilian propolis effects on growth,
Kylymnyuk, O.; Khimich, O.; Laptieyev, O. — Propolis in the diet of broiler chickens as a natural sourc
Quishpe Mendoza, X. C.; Molina Molina, E. J.; Armas Cajas, J. W.; Mera Viera, E. H. — Use of propolis
Martínez, D. A.; Ponce-de-León, C. L.; Vilchez, C. — Effects of phytogenic feed additives including pro
Kairalla Sebha, M. — Effects of propolis powder on growth performance, physiological traits and car
Cristóbal Quishpe Mendoza, X. et al. — Propolis extract supplementation effects on immunity, blood
Martinez, D. — Oregano and phytogenic additives including propolis on intestinal health and product
Chuy, M.; Cumpa, M.; Martinez, D. — Effect of antimicrobial feed additives on broiler performance a
Li, J.; Kim, I. H. — Effects of Saccharomyces cerevisiae cell wall extract and poplar propolis ethanol ex
Khalil, M. L.; et al. — Effects of propolis supplementation on growth performance and immune respo
Ghosh, S.; et al. — Ethnoveterinary use of propolis in pig production systems: antimicrobial and healt
Zhang, Y.; Wang, L.; et al. — Dietary propolis improves gut microbiota and growth performance in we
Valpotić, H.; et al. — Biological and immunomodulatory effects of propolis in pigs and other farm ani
Manav, S.; Yilmaz, M.; Baytekin, H.; Çelik, K. — Propolis as a natural antimicrobial feed additive in live
De Agüero, M. L.; et al. — Propolis as a natural feed additive in animal production systems including
Abu-Seida, A. M. — Potential benefits of propolis in veterinary medicine including pig production: na
Santos, L. M.; Fonseca, M. S.; Sokolonski, A. R.; et al. — Propolis: biological activities and veterinary a
Falcão, S. I.; et al. — Chemical variability and biological activity of propolis with relevance to animal p
Papaioannou, D. S.; Kyriakis, S. C.; et al. — Phytogenic feed additives including propolis in pigs: effect
Naseem, R.; Khan, M. N.; et al. — Effect of natural zeolite and propolis-related compounds on nutrie
Ito, M.; et al. — Raw propolis supplementation and growth performance in weanling pigs — 2012
Haščík, P.; et al. — Effects of propolis supplementation in animal diets: comparative livestock respon
Perina, J.; et al. — Feed additive effects including propolis in swine production under controlled conc
Sbardella, M.; et al. — Non-antibiotic feed additives in pig nutrition: role of phytogenics including pro
Yu, et al. — Propolis-based feed additives and growth performance in nursery pigs — 2024
Gois, F. D.; et al. — Evaluation of natural additives including propolis in swine diets — 2016
Niewold, T. A. — The non-antibiotic effects of feed additives including propolis in pig intestinal healt
Roura, E.; et al. — Phytogenic compounds including propolis in pig nutrition: mechanisms of action –
Lima de Souza, A. K.; Colares, R. R.; Lima de Souza, A. C. — The main uses of ozone therapy in disease
Sumida, J. M.; Hayashi, A. M. — Ozone therapy in veterinary medicine: clinical indications and techni
Botacini, S. B.; et al. — Ozone therapy in veterinary medicine – Literature review — 2024
Giménez, B.; Zaritzky, N. E.; Graiver, N. G. — Ozone treatment of meat and meat products: a review
Novotný, J.; Reichel, P.; Bárdová, K.; et al. — Effects of clinoptilolite administration on intestinal heal
Valpotić, H.; et al. — Zeolite clinoptilolite nanoporous feed additive: potentials and limitations in ani
Maity, S.; Rubić, I.; et al. — Clinoptilolite supplementation restores energy balance in dairy cows: om
Abdelrahman, M. M.; Alhidary, I. A.; et al. — Clinoptilolite effects on mineral metabolism in growing
Zhai, H.; Liu, H.; Wang, S.; et al. — Essential oils and plant extracts in pig nutrition: gut health effects
Abd El-Hack, M. E.; Alagawany, M.; et al. — Phytogenic feed additives in poultry nutrition: a review –
Alhidary, I. A.; et al. — Plant secondary compounds and performance in sheep and goats — 2021
Diaz-Sanchez, S.; D'Souza, D.; et al. — Botanicals in poultry: applications and mechanisms — 2015
Santos, L. M.; Fonseca, M. S.; Sokolonski, A. R.; et al. — Propolis: biological activities and veterinary a
Stevanović, Z. D.; Glavinić, U.; et al. — Bee-inspired healing: apitherapy in veterinary medicine — 202
Manav, S.; Yilmaz, M.; Baytekin, H.; et al. — Propolis as antimicrobial feed additive in livestock — 202

Abu-Seida, A. M. — Propolis in large and small animal veterinary practice: narrative review — 2023
Lima de Souza, A. K.; Colares, R. R.; Lima de Souza, A. C. — The main uses of ozone therapy in disease
Botacini, S. B.; et al. — Ozone therapy in veterinary medicine: literature review — 2024
Sumida, J. M.; Hayashi, A. M. — Ozone therapy in veterinary medicine: clinical indications and techni
Bocci, V. — Ozone: a new medical drug — 2011
Re, L.; Martínez-Sánchez, G.; et al. — Ozone therapy: a clinical review of mechanisms and applicatior
Elvis, A. M.; Ekta, J. S. — Ozone therapy: a clinical review — 2011
Novotný, J.; Reichel, P.; Bárdová, K.; et al. — Effects of clinoptilolite administration on intestinal heal
Valpotić, H.; et al. — Zeolite clinoptilolite nanoporous feed additive: potentials and limitations in ani
Katsoulos, P. D.; et al. — Clinoptilolite supplementation and metabolic effects in dairy cows — 2015
Dschaak, C. M.; Eun, J. S.; et al. — Effects of natural zeolite on dairy cow performance and ruminal fe
Maity, S.; Rubić, I.; et al. — Clinoptilolite supplementation restores energy balance in dairy cows — 2
Abdelrahman, M. M.; Alhidary, I. A.; et al. — Clinoptilolite effects on mineral metabolism in goats —
Zhai, H.; Liu, H.; Wang, S.; et al. — Essential oils and plant extracts in pig nutrition — 2018
Abd El-Hack, M. E.; Alagawany, M.; et al. — Phytogenic feed additives in poultry nutrition — 2016
Diaz-Sanchez, S.; D'Souza, D.; et al. — Botanicals in poultry production — 2015
Windisch, W.; Schedle, K.; Plitzner, C.; Kroismayr, A. — Use of phytogenic products as feed additives
Santos, L. M.; Fonseca, M. S.; Sokolonski, A. R.; et al. — Propolis: biological activities and veterinary a
Kurek-Górecka, A.; Rzepecka-Stojko, A.; et al. — Propolis in veterinary medicine: antimicrobial and in
Sforcin, J. M.; Bankova, V. — Propolis: is there a potential for veterinary use? — 2011
Bankova, V. — Recent trends in propolis research — 2012
Stevanović, Z. D.; Glavinić, U.; et al. — Bee-inspired healing: apitherapy in veterinary medicine — 202
Abu-Seida, A. M. — Propolis in large and small animal veterinary practice — 2023
Laurino, C.; Palmieri, B. — Propolis as a therapeutic agent in veterinary medicine — 2015
Manav, S.; Yilmaz, M.; Baytekin, H.; et al. — Propolis as antimicrobial feed additive in livestock — 202
Lima de Souza, A. K.; Colares, R. R.; Lima de Souza, A. C. — The main uses of ozone therapy in large a
Botacini, S. B.; et al. — Ozone therapy in veterinary medicine: updated literature review — 2024
Sumida, J. M.; Hayashi, A. M. — Ozone therapy in veterinary clinical practice: indications and protocc
Stevanović, Z. D.; Glavinić, U.; et al. — Bee products in veterinary medicine: apitherapy update — 20
Santos, L. M.; Fonseca, M. S.; Sokolonski, A. R.; et al. — Propolis: biological activities and veterinary a
Abu-Seida, A. M. — Propolis in large and small animal veterinary practice: narrative review — 2023
Manav, S.; Yilmaz, M.; Baytekin, H.; et al. — Propolis as antimicrobial feed additive in livestock: overv
Falcão, S. I.; et al. — Chemical variability and biological activity of propolis in animal health — 2019
Maity, S.; Rubić, I.; et al. — Clinoptilolite supplementation and metabolic recovery in dairy cows: omi
Valpotić, H.; et al. — Zeolite clinoptilolite as feed additive in animal production: potentials and limita
Novotný, J.; Reichel, P.; Bárdová, K.; et al. — Clinoptilolite effects on intestinal health in pigs — 2019
Abdelrahman, M. M.; Alhidary, I. A.; et al. — Clinoptilolite and mineral metabolism in small ruminant
Zhai, H.; Liu, H.; Wang, S.; et al. — Essential oils and plant extracts in pig nutrition: gut health effects
Abd El-Hack, M. E.; Alagawany, M.; et al. — Phytogenic feed additives in poultry nutrition: a review –
Diaz-Sanchez, S.; D'Souza, D.; et al. — Botanicals in poultry production systems — 2015
Windisch, W.; Schedle, K.; Plitzner, C.; Kroismayr, A. — Phytogenic feed additives in animal nutrition
Laurino, C.; Palmieri, B. — Propolis as a therapeutic agent in veterinary medicine — 2015
Kurek-Górecka, A.; Rzepecka-Stojko, A.; et al. — Propolis in veterinary medicine: antimicrobial and in
Sforcin, J. M.; Bankova, V. — Propolis: potential in veterinary and biomedical use — 2011
Bankova, V. — Advances in propolis research and applications — 2012
Abu-Seida, A. M. — Veterinary applications of propolis in animal health — 2023

tibiotic alternatives in animal health and production: A review of the literature of the last decade. Animal nutriti
omoters in Livestock: A Scoping Review. Agriculture 2026, 16, 559. https://doi.org/10.3390/agriculture16050559

policy, and potential. Public health reports, 2012, 127(1), 4–22. https://doi.org/10.1177/003335491212700103

Viallouris, A.; Filippou, C.; Johnson, E.O. Antibiotic Use in Livestock Farming: A Driver of Multidrug Resistance? Mi

ja, M. Effects of dietary clinoptilolite supplementation on β-hydroxybutirate serum level and milk fat to protein r
oxidative biomarkers in dairy cows. Res. Vet. Sci. 2019a, 127, 57–64. https://doi.org/10.1016/j.rvsc.2019.10.010
Habrun, B.; Njari, B.; Kovšca Janjatović, A.; Efendić, M.; Samardžija, M.; Popović, M.; Valpotić, I.; Špoljarić, B. Imm
sić, N.; et al. Integrated metabolomics and proteomics dynamics of serum samples reveals dietary zeolite clinopti
Blazi, M., Krizanac, S., Stojković, R., Jurin, M., Subotić, B., & Colić, M. Natural zeolite clinoptilolite: new adjuvant i
N.; et al. Zeolite clinoptilolite nanoporous feed additive for animals of veterinary importance: Potentials and lim
fficacy on the reduction of milk aflatoxin M1 concentration in dairy cattle. J Anim Sci Technol, 2016, 58, 24. https
alpotić, I. In-feed supplementation of clinoptilolite favourably modulates intestinal and systemic immunity and sc
ance in Beef Steers Fed High Concentrate Diets, Journal of Animal Science, 1983, 56(3), 517–524, https://doi.org
in a Semicontinuous In Vitro System. Animals, 2022, 12(3), 345. https://doi.org/10.3390/ani12030345
arda, T.Y.; Dávila-Ramírez, J.L.; Valenzuela-Melendres, M.; González-Rios, H. Ferulic Acid and Clinoptilolite Affect

ma, M.; Mota-Rojas, D.; Sherasiya, A.; Ciani, F.; El-Sabrout, K. Bee Products for Poultry and Rabbits: Current Chal
nst clinically relevant multidrug-resistant bacteria. Front. Microbiol., 2024, 15, 1480433. doi: 10.3389/fmicb.202
130, 240–246. https://doi.org/10.1016/j.rvsc.2020.03.026Abdelnour, S.A.; Abd El-Hack, M.E.; Alagawany, M.; Fa

lication on reproductive efficiency in Holstein cows. Reprod. Domest. Anim. 2012, 47, 87–91. https://doi.org/10.
stitis, metritis and retention of fetal membranes in the cow. Proceedings of the 2nd International Symposium on

džija, M. Effects of dietary clinoptilolite supplementation on udder health and chemical composition of milk in d

nedical use in sheep with retained placenta. Reprod. Domest. Anim. 2016, 51, 538–540. https://doi.org/10.1111/

mardžija, M. Shortening days open using intrauterine ozone therapy in Simmental cows. In Veterinarska stanica,
r, Z.; et al. Effects of dietary clinoptilolite on reproductive performance, serum progesterone and insulin-like gro

of North America. Small animal practice, 2025, 55(6), 1117–1136. https://doi.org/10.1016/j.cvsm.2025.06.009

ć, I.; Šuran, J. Intramammary propolis formulation for subclinical mastitis prevention and treatment in dairy cow
ne antioxidant effect of the novel bee-product based intramammary formulation Apimast® in dairy cattle. Veteri

F. Antimicrobial properties of hive products and their potential applications in human and veterinary medicine.
Hellenic Veterinary Medical Society, 2022, 73(2), 3905–3912. https://doi.org/10.12681/jhvms.26334
oultry and Rabbits: Current Challenges and Perspectives. Animals 2023, 13, 3517. https://doi.org/10.3390/ani13

:s: Potentials and Challenges in Application. Pathogens 2015, 4, 137-156. https://doi.org/10.3390/pathogens401
C.; González-Olivares, L.G.; Baena-Santillán, E.S.; Ocampo-Salinas, I.O.; Guerrero-Solano, J.A.; et al. Plant Antimic

s in livestock: Impacts on health and production. Animals 2021, 11, 140. https://doi.org/10.3390/ani11010140
view of plant-derived essential oils in ruminant nutrition and production. Animal Feed Science and Technology, 2
saponins on the production performance, serum biochemical factors, and immune factors in Small-Tailed Han s

of Medicinal Plants as Natural Additives and Anthelmintics in Ruminant Diets: A Systematic Review. Animals 202
npalam, E. N. Phytochemicals in Ruminant Diets: Mechanistic Insights, Product Quality Enhancement, and Pathwa

s for Different Farm Animals Under European Conditions. Frontiers in veterinary science, 2018, 5, 140.

alt-tolerant plants: Exploring its potential use as fodder, nutraceuticals or phytotherapeutics in ruminant produc
ith medicinal plants: a systematic review of European ethnoveterinary research. Forschende Komplementarmed

enhorst, M. What can we learn from past and recent Bavarian knowledge for the future development of Europea
enlacher, T. Farmers' knowledge in the Swiss canton Valais: cultural heritage with future significance for Europea
ation by indigenous and local communities of Dugda District, Central Rift Valley, Ethiopia. J Ethnobiology Ethnom
in the Omo-Gibe and Rift Valley basins of Ethiopia. BMC Vet. Res. 2024, 20, 166. doi:10.1186/s12917-024-04019
of standardized ginger extract on gut morphology and performance in broilers. Agriculture 2025, 15, 1448. http
d in Uganda: Their phytochemistry, bioactivity and toxicity. J. Ethnopharmacol. 2026, 358, 120917. doi:10.1016/
jkić, M.; et al. The influence of dietary clinoptilolite on blood serum mineral profile in dairy cows. Vet. Arhiv 2019
eatment of puerperal disorders in domestic ruminants: A review. Vet. Arhiv 2017, 87, 363–375. https://doi.org/1

e, zeolite, and propolis in domestic ruminants. In XXXIII International Scientific Congress of the Hungarian Associ
o, G. Intrauterine oxygen/ozone mixture for the treatment of subclinical endometritis in repeat breeder cows. R
uterine therapy with ozone reduces subclinical endometritis and improves reproductive performance in postpar

Pirone, A., Lazzarini, G., Passamonti, F., Marmorini, P., Ori, M., & Panzani, D. (2026). Effects of intrauterine ozor
o Conventional Antibiotics and Antiseptics. Vet Sci., 2024, 11(4), 163 doi:10.3390/vetsci11040163Orlandin, J.R.;

obial activity of brown propolis ethanolic extract and its application in intramammary formulation for the treatm
azinskiene O and Trumbeckaite S. Propolis as an alternative remedy for the treatment of subclinical mastitis in d
nese propolis in protecting bovine mammary epithelial cells against mastitis pathogens-induced cell damage. Me
Manuka honey gel on the transforming growth factor β1 and β3 concentrations, bacterial counts and histomorph
J.; Papazoglou, L.G. Evaluation of the effectiveness of medical-grade honey and Hypericum perforatum ointment

ee venom supplementation in drinking water on growth performance of broiler chickens. Poult. Sci. 2010, 89, 23

and Zeoula, L.M. Effects of phenolic compounds in propolis on digestive and ruminal parameters in dairy cows. R

Portela, R.D. and Machado, B.A. Propolis: types, composition, biological activities, and veterinary product patent
ealing: Apitherapy in Veterinary Medicine for Maintenance and Improvement Animal Health and Well-Being. Pha

tibacterial, Antifungal, and Antiparasitic Properties of Propolis: A Review. Foods 2021, 10, 1360. https://doi.org/
m Medicago spp. against sheep gastrointestinal nematodes. Molecules, 2020, 25(2), 242. https://doi.org/10.339
ve agent in poultry industry. World's Poult. Sci. J. 2017, 73, 483–492. https://doi.org/10.1017/S0043933917000

(L) DC, a traditional herbal medicine, reduces inflammation, oxidative stress and protects the intestinal barrier in
enom-derived antimicrobial peptide, may target methicillin-resistant Staphylococcus aureus. Mol. Med. Rep. 201

l Chinese medicine on antioxidative status and inflammatory cytokines expression in the liver of piglets. Front. V
ions: Phytogenic Alternatives and Effective Monitoring; the Dutch Approach. Front. Vet. Sci., 2021, 8, 709750. dc
apy. Frontiers in veterinary science, 2023, 10, 1171987. https://doi.org/10.3389/fvets.2023.1171987

herbal medicine compound on coronavirus disease 2019 (COVID-19): a randomized controlled trial. Integr. Med

th Medicinal Plants: A Systematic Review of European Ethnoveterinary Research. Forsch. Komplementärmed. 20
Valkenhorst, M. Swiss ethnoveterinary knowledge on medicinal plants – a within-country comparison of Italian s

based remedies to treat cattle, pigs, horses, and other domestic animals in the Mediterranean island of Sardinia

ian Peninsula). In Herbs, Humans and Animals / Erbe, Uomini e Bestie; Pieroni, A., Ed.; Experiences Verlag: Colog
alonia and the Balearic Islands. Evid.-Based Complement. Altern. Med. 2012, 896295. https://doi.org/10.1155/2

A.; Volpato, G.; Sõukand, R. Multifarious Trajectories in Plant-Based Ethnoveterinary Knowledge in Northern and

e Arribes del Duero, western Spain. J Ethnopharmacol. 2010;131:343-355. doi: 10.1016/j.jep.2010.07.022.

in traditional veterinary practices in Andalusia. In Herbs, Humans and Animals; Pieroni, A., Ed.; Experiences Verla
ook: Forrages and grazing in equine nutritionEdition: EAAP Scientific Series, Vol. 132Chapter: Part 9Publisher: Wa

ary Medicine in Central Anatolia Region. Turk. J. Agric. Food Sci. Technol. 2017, 5(13), 1690-1695. https://doi.org

siotis, K.; Gardikis, K.; Dias, P.; Oluški, M.; Muñoz Montaño, J.R.; Hristova, H.; Iliev, H.; Petrangolini, G.; Afantitis,

ey sources of traditional knowledge: a quantitative ethnobotanical survey in the central Balkans. Journal of ethno

, V.P.S. Ethnoveterinary knowledge in Pirot county (Serbia). S. Afr. J. Bot. 2021, 137, 278–289. doi: 10.1016/j.sajb
nt knowledge among Albanians,Macedonians and Gorani in the Sharr Mountains (Republic of Macedonia). Gene

nts in the Curvature Subcarpathians area, Romania. Scientific Works. Series C. Veterinary Medicine 2020, 66, 93-
Coast Region (Bulgaria) -Ethnobotanical Research. RJPBCS 2021, 11, 83-95. DOI: 10.33887/rjpbcs/2020.11.6.11
thnoveterinary knowledge in Swiss alpine regions. J. Ethnopharmacol. 2019, 234, 225–244. https://doi.org/10.1
M. Ethnoveterinary knowledge of farmers in bilingual regions of Switzerland. J. Ethnopharmacol. 2020, 246, 112
horst, M. What can we learn from past and recent Bavarian knowledge for the future development of European
horst, M. What can we learn from past and recent Bavarian knowledge for the future development of European

py with ozone, zeolite, and propolis in domestic ruminants // XXXIII International Scientific Congress of the Hung
Damjan ; Đuričić, Dražen Intrauterine ozone treatment of puerperal disorders in domestic ruminants: a review /
ntibiotics versus ozone medical use in sheep with retained placenta and following obstetric assistance // Reprod
estic ruminants // Proceedings of XXV International Congress of the Hungarian Assocition for Buiatrics. Budimpe
OMETRITIS IN DAIRY COWS // INNOVATIVE PROCESSES IN AGRO-INDUSTRIAL COMPLEX. Moskva: Российский уı
arko Primjena ozona u bujatrici // Veterinarski dani 2015. : Zbornik radova / Zagreb : Hrvatska veterinarska kom
in buiatrics : Current knowledge // Animal reproduction science, 159 (2015), 1-7. doi: 10.1016/j.anireprosci.201
etal Membrane in Dairy Goats by Ozone : Novel Alternative to Antibiotic Therapy // Reproduction in domestic aı
uričić, Dražen Effect of intrauterine ozone treatment in cows with repeat-breeding syndrome after bacteriologica
SOBNOST KRAVA SIMENTALSKE PASMINE / Samardžija, Marko (mentor). Zagreb, Veterinarski fakultet, Zagreb, 2(

lajsig, Berislav ; Plavec, Helena ; Smolec, Ozren Intrauterine ozone administration for improving fertility rate in S
nislav OZONE THERAPY OF RETAINED PLACENTA IN HOLSTEIN COWS // XIII Middle European Buiatric's Congress
TI KOD KRAVA // Petnaesto regionalno savetovanje iz kliničke patalogije i terapije životinja. Beograd: Fakultet ve
ozone treatment of retained fetal membrane in Simmental cows // Animal reproduction science, 134 (2012), 3/
l Treatment Option for Urovagina in Dairy Cows // Reproduction in domestic animals, 47 (2012), 2; 293-298. doi
eventive Intrauterine Ozone Application on Reproductive Efficiency in Holstein Cows // Reproduction in domestic
Treatment Option for Urovagina in Dairy Cows // 19th International Congress of Mediterranean Federation of H
anović, Nikica ; Folnožić, Ivan ; Smolec, Ozren ; Samardžija, Marko Shortening Days Open Using Intrauterine Ozo

s against Chlamydophila felis in cats positive to FIV and/or FELV // Acta veterinaria (Beograd), 58 (2008), 1; 17-23
lis in cats with immunocompromised syndrome // Proceedings of the Fourth Workshop COST Action 855 - Diagn
Blanka ; Turk, Romana ; Gračner, Damjan ; Maćešić, Nino et al. Integrated Metabolomics and Proteomics Dynar
er, Vesna ; Đidara, Mislav Selenium and natural zeolite clinoptilolite supplementation increases antioxidative sta
čner, Damjan ; Maćešić, Nino ; Folnožić, Ivan ; Šostar, Zvonimir et al. Effects of dietary clinoptilolite on reproducti
n ; Dobranić, Tomislav ; Benić, Miroslav ; Samardžija, Marko Effects of Dietary Vibroactivated Clinoptilolite Supp
a ; Maćešić, Nino ; Benić, Miroslav ; Getz, Iva ; Samardžija, Marko Effects of dietary clinoptilolite supplementatio
potić, Hrvoje ; Beer Ljubić, Blanka ; Lojkić, Martina ; Gračner, Damjan et al. Effects of in-feed clinoptilolite treatm
otić, Hrvoje ; Gračner, Damjan ; Maćešić, Nino ; Lojkić, Martina et al. The influence of dietary clinoptilolite on blo
ožić, Ivan ; Grizelj, Juraj ; Getz, Iva ; Šostar, Zvonimir et al. Modulating effects of dietary clinoptilolite (CPL) on pr
ožić, Ivan ; Grizelj, Juraj ; Getz, Iva ; Šostar, Zvonimir et al. Modulating effects of dietary clinoptilolite (CPL) on pr
obranić, Vesna ; Getz, Iva ; Lojkić, Martina ; Samardžija, Marko Effects of dietary clinoptilolite supplementation
lušić, Sanja ; Turk, Nenad ; Šostar, Zvonimir ; Samardžija, Marko Acute phase response and oxidative stress in pe
v ; Gračner, Damjan ; Dobranić, Tomislav ; Lojkić, Martina ; Vince, Silvijo et al. Influence of dietary clinoptilolite s
en ; Maćešić, Nino ; Mikulec, Željko ; Kočila, Predrag ; Sobiech, Przemyslaw et al. Dietary supplementation with r
mjan ; Starič, Jože ; Cvetnić, Luka ; Folnožić, Ivan Effect of dietary clinoptilolite supplementation on somatic cell c
ojkić, Martina ; Žura Žaja, Ivona ; Bedrica, Ljiljana ; Maćešić, Nino et al. Zeolite clinoptilolite nanoporous feed add
; Cvetnić, Luka ; Gračner, Damjan ; Vince, Silvijo ; Grizelj, Juraj et al. Dietary zeolite clinoptilolite supplementatio
randa, Marcela ; Žura Žaja, Ivona ; Đuričić, Dražen ; Bach, Ana ; Harapin, Ivica et al. Influence of dietary mannan c

oslav ; Vince, Silvijo ; Cvetnić, Luka ; Sadiković, Mirsad Effects of dietary zeolite clinoptilolite on health and reproc
lana ; Habrun, Boris ; Đuričić, Dražen ; Sadiković, Mirsad ; Valpotić, Ivica In-feed supplementation of clinoptilolite
renka ; Smodis Skerl, Ivana Maja Zeolite clinoptilolite as a dietary supplement and remedy for honeybee (Apis me
ka ; Mihelić, Damir ; Kozačinski, Lidija ; Popović, Maja Chemical evaluation of the quality of meat of broilers fed w
lec, Željko ; Habrun, Boris ; Špoljarić, Daniel Influence of natural zeolite clinoptilolite on immunological paramete
terial counts in pig slurry // Proceedings of the 3rd Croatian-Slovenian Symposium on zeolites. Zagreb: CROZA, 20
Herzig, Ivan Effect of Feed Additive Clinoptilolite (ZeoFeed) on Nutrient Metabolism and Production Performance
of a clinoptilolite-supplemented feed to layers and its effect on performance, haematological parameters and m
: experimental evidence of the molecular mechanisms accounting for its therapeutic action." International Journ

aunoras, G. (2024). A comparative study of the chemical properties and antibacterial activity of four different ozo
herapeutic potential of ozone water treatment in alleviating atopic dermatitis symptoms in mouse models: Explo
mical and Antimicrobial Evaluation of Ozonated Olive Oil Produced with a Medical-Grade Generator for Veterina
on of antibacterial oxygen/ozone mixture in vitro activity on bacteria isolated from cervico-vaginal mucus of cov
., ... & Estrela-Lima, A. (2024). Ozone therapy in the integrated treatment of female dogs with mammary cancer
& Ambrósio, C. E. (2023). Ozone therapy: protocol for treating canine parvovirus infection. Brazilian journal of ve
F. (2022). In vitro porphyrin-based photodynamic therapy against mono and polyculture of multidrug-resistant b
ro effects of different ozone preparations on microorganisms responsible for endometritis in the mare. Therioge
rison between ozone therapy and electroacupuncture for canine thoracolumbar disk disease. Research in Veteri
in vitro effect of ozone therapy against equine Pythium insidiosum. Journal of Equine Veterinary Science, 98, 103
ety and effects of a commercial ozone foam preparation on endometrial environment and fertility of mares. Jou
y as an alternative to conventional antibiotics and antiseptics. Veterinary Sciences, 11(4), 163.

activity of ozone/oxygen gaseous mixture against a caprine herpesvirus type 1 strain isolated from a goat with v
essely-Szponder, J. (2026). Mild Ozone-Induced Oxidative Stress Modulates the Activity and Viability of Porcine
026). Protective Effects of Ozone against Quinolinic Acid-Induced Redox Imbalance in Murine BV-2 Microglial Cel
). Uso de ozonioterapia no tratamento de feridas em cão: Relato de caso. Pubvet, 16(10), e1242-e1242.
auterine oxygen/ozone mixture for the treatment of subclinical endometritis in repeat breeder cows. Research i
de Oliveira, V. A. P., Pinto, I

. In Vitro Effects of PRP, Ozonized PRP, Hyaluronic Acid, Paracetamol, and Polyacrylamide on Equine Synovial Flu

reira, D. I. B. (2024). In vitro and ex vivo anti-Pythium insidiosum potential of ozonated sunflower oil. Brazilian Jo
. D. M., & Araújo, P. R. M. (2026). Integrative Therapies in Wound Healing in Small Animals: An Approach Beyon
oncentrations promote in vitro preservation of explanted articular cartilage: an ultrastructural study. European
ozonioterapia no tratamento de dermatite úmida aguda em cães: Relato de três casos. Pubvet, 17(04), e1370-e

, & Nogueira-Filho, S. L. G. (2026). Assessment of Blood Profiles and Pain in Sport Horses Undergoing Intrarectal
. & Escodro, P. B. (2026). Validation of the use of ozonated water in sanitization and prevention of mastitis in da
, J. C. (2024). Effectiveness of photo-ozone therapy against equine Pythium insidiosum. Journal of Equine Veterin
o evaluation of ozonated water treatment on the viability of eimeria oocysts and giardia cysts from water buffalo
nical responses of rectal oxygen-ozone administration in sheep. Veterinary Research Communications, 50(4), 26
A. O. (2025). Bovine mastitis mycotic cause, updates in diagnostic and therapeutic tools, and economic impacts o
nsumer preferences: changing trends and the way forward. Journal of Culinary Science & Technology, 21(5), 719-
EQUENOS ANIMAIS: REVISÃO. COMPARATIVE AND TRANSLATIONAL MEDICINE Учредители: Instituto Addere, 1(

tion of the Healing Effects of Different Methods of Using Ozone in Third-Degree Skin Burns in Rat Experimental M

... & Першина, К. С. (2026). ОЗОНОТЕРАПИЯ В СОВРЕМЕННОЙ МЕДИЦИНЕ: МЕХАНИЗМЫ ДЕЙСТВИЯ, КЛИНИ
scodro, P. B. (2022). Effects of ozone therapy on hematological, biochemical, and oxidative stress parameters of
In vitro and in vivo safety and efficacy of ozonized olive oil for the treatment of naturally occurring acute clinical

la biometría y bioquímica hemática en caballos clínicamente sanos. Revista de Medicina Veterinaria, (45), e0005

ernativos aos convencionais com solução fisiológica ozonizada em fêmeas bovinas lactantes com endometrite cl
d adipose tissue mesenchymal stem cells (AT-MSCs) with Ozone therapy for treating articular cartilage defect of

tia toracolombar: estudo prospectivo randomizado e cego (Doctoral dissertation, Universidade de São Paulo).
, J., Garcia, M. E., ... & Portero Fuentes, M. The effect of ozonated oil on the treatment of chronic vaginitis in fem
: Revisão de Literatura Efficacy of Intra-Articular Ozone Therapy in Canine Osteoarthritis: Literature Review.

SMA INTRA-UTERINE THERAPY IN INFECTIOUS REPEAT BREEDER COWS. Indian Journal of Animal Reproduction, 4
ВАННЯ СТАБІЛІЗОВАНОГО ВОДНОГО ОЗОНУ ДЛЯ САНАЦІЇ БІОАЕРОЗОЛЮ ТА ПОВЕРХОНЬ У КЛІНІКАХ ВЕТЕР

... & Mylostyvyi, R. V. (2021). Ефективність озонотерапії кіз із маститом. Theoretical and Applied Veterinary M

terino liječenje puerperalnih poremećaja domaćih preživača ozonom-pregledni članak. Veterinarski arhiv, 87(3),

ne, K. (2024). The comparative study of the antioxidant and antibacterial effects of propolis extracts in veterinar

propolis against bacteria, yeast, and trichomonas gallinae isolated from pigeons—A possible antibiotic alternati

, C. (2002). A field study on the effect of in-feed inclusion of a natural zeolite (clinoptilolite) on health status and

ural alternatives: in vitro efficacy of Hungarian propolis against feline and bovine Tritrichomonas foetus. Frontier

obiotic efficacy in rabbits challenged with multidrug-resistant Escherichia coli. Journal of Advanced Veterinary Re

Mitigation of Methane Production in Ruminants. International Journal of Research and Development in Pharmac

H.; Fernández-Montequín, J. I.; León, O. S. — *Therapeutic efficacy of ozone in patients with diabetic foot* — 2015

er-soluble fraction of bee venom produces antinociceptive and anti-inflammatory effects in animal models — 20

ho, A. A.; Padilha, F. F.; Barbosa, J. D. V.; Umsza-Guez, M. A. — Antioxidant, antimicrobial, antiparasitic, and cyto

I. — Molecular mechanism underlying anti-inflammatory and anti-allergic activities of phytochemicals in propolis

Sassaki, G. L.; Iacomini, M.; Otuki, M. F. — Topical anti-inflammatory activity of a monofloral honey in animal mo

.; Ghramh, H. A.; Adgaba, N.; Dash, C. K. — Composition and functional properties of propolis (applications in an

arag, M. R.; Alam, G. M. — Multiple beneficial applications and modes of action of herbs in poultry health and pr

ınd natural zeolite clinoptilolite supplementation increases antioxidative status and immune response in growing

ıpplementation with clinoptilolite on performance and selected serum biochemical values in dairy goats — 2009

ı performance, and carcass of sheep fed urea-based diet supplemented with natural clinoptilolite — 2022

olite supplementation restores energy balance in high-yielding dairy cows: integrated metabolomics and proteor

ral zeolite on intake, digestion, ruminal fermentation, and lactational performance of dairy cows — 2010

s extract against Staphylococcus coagulase positive and Malassezia pachydermatis of canine otitis — 2010

‹tract supplementation on growth performance, digestibility, blood profile, fecal microbiota and fecal noxious ga

ratio during early lactation in Holstein-Friesian cows. Micropor. Mesopor. Mater. 2020a, 292, 109766. https://do

unogenicity of a Live Bivalent Non-Enterotoxigenic Escherichia coli (Non-ETEC) Vaccine and Dietary Clinoptilolite
ilolite supplementation restores energy balance in high yielding dairy cows. Metabolites 2021, 11, 842. https://d
n anticancer therapy. Journal of molecular medicine (Berlin, Germany), 2001, 78(12), 708–720. https://doi.org/1

In Vitro Rumen Fermentation Characteristics and Bacterial Abundance. Fermentation 2024, 10, 549. https://doi

arag, M.R.; Elnesr, S.S. Beneficial impacts of bee pollen in animal production, reproduction and health. J. Anim. Pl

Ozone Applications, Havana, Cuba. 1997, 1, 35-37Abo-El-Sooud K. Ethnoveterinary perspectives and promising f

airy cows over two consecutive years. In Abstract Book of the 30th World Buiatric Congress; Japanese Society of

owth factor-1 concentrations in dairy cows during pregnancy and lactation. Pol. J. Vet. Sci. 2020, 23, 69–75. https

robial Compounds and Their Mechanisms of Action on Spoilage and Pathogenic Bacteria: A Bibliometric Study ar

ays to Sustainable Milk and Meat Production-Invited Review. Animals, 2026, 16(3), 425. https://doi.org/10.3390

n veterinary herbal medicine? An ethnoveterinary study. Journal of ethnopharmacology, 2022, 288, 114933.
n veterinary medicine?. Journal of ethnobiology and ethnomedicine, 2024, 20(1), 73. https://doi.org/10.1186/s

tum dairy cows managed in pasture-based systems. Trop Anim Health Prod, 2020, 52, 2523–2528 https://doi.or

ıe insufflation in eleven subfertile mares: a case series. Journal of equine veterinary science, 105778 . https://do
Machado, L.C.; Ambrósio, C.E.; Travagli, V. Ozone and its derivatives in veterinary medicine: A careful appraisal. '

ent of bovine mastitis. Brazilian journal of microbiology, 2026, 57(1), 30. https://doi.org/10.1007/s42770-025-01

nology of contaminated full-thickness skin wounds in equine distal limbs. Aust. Vet. J., 2016, 94, 27–34.

evista Brasileira de Zootecnia, 2014, 43, 197-206. https://doi.org/10.1590/S1516-35982014000400006

a murine model of colitis. Inflammopharmacology 2020, 28, 1717–1734. https://doi.org/10.1007/s10787-019-0

peaking regions with north-western German speaking regions. J. Ethnobiol. Ethnomed. 2017, 13, 1. https://doi.o

ageningen Academic Publishers (Eds. Saastamoinen, M.; Fradinho, M.J.; Santos, A.S.; Miraglia N.). EAAP Scientific

A.; Aligiannis, N. EthnoHERBS: Harnessing traditional herbal knowledge for biodiversity conservation and innova

veterinary herbal medicine? An ethnoveterinary study. Journal of Ethnopharmacology 2022, 288, 114933. https
veterinary herbal medicine? An ethnoveterinary study. Journal of Ethnopharmacology 2022, 288, 114933.

- Congress proceedings. Beograd: Serbian Buiatric's Association, Faculty of Veterinary Medicine, University of Be

ealth and Production of Ruminants, Belgrade, Serbia. Beograd: Univerzitet u Beogradu, 2011. str. 475-480
ne Therapy in Simmental Cows // Veterinarska stanica, Supplement 1, Book of Proceedings. Zagreb, 2011. str. 1

nics of Serum Samples Reveals Dietary Zeolite Clinoptilolite Supplementation Restores Energy Balance in High Yi
tus and immune response in growing pigs // Frontiers in veterinary science, 8 (2021), 688915, 10. doi: 10.3389/f
ive performance, serum progesterone and insulin-like growth factor-1 concentrations in dairy cows during pregn
lementation on the Intramammary Microbiological Findings in Dairy Cows // Animals, 10 (2020), 2; 202, 8. doi: 1
n on β-hydroxybutirate serum level and milk fat to protein ratio during early lactation in Holstein-Friesian cows /
ıent on serum metabolic and antioxidative biomarkers and acute phase response in dairy cows during pregnancy
od serum mineral profile in dairy cows // Veterinarski arhiv, 89 (2019), 4; 447-462. doi: 10.24099/vet.arhiv.0662
ogesterone (P4) and insulin-like factor 1 (IGF-1) blood concentrations in Holestein-Friesian cows during pregnanc
ogesterone (P4) and insulin-like growth factor 1 (IGF-1) blood concentrations in Holstein-Friesian cows during pr
on udder health and chemical composition of milk in dairy cows over two consecutive years // Abstract book of t
riparturient dairy cows supplemented with zeolite clinoptilolite // Proceedings of the 18th Biennial Congress of t
supplementation on milk fat to protein ratio and β-hydroxybutirate serum levels during transition period in dairy
nannan oligosaccharide and clinoptilolite modulates innate and adaptive immune parameters of weaned pigs //
counts, udder health and chemical composition of milk in Holstein-Frisian cows // 27th International Congress of
litive for animals of veterinary importance: potentials and limitations // Periodicum biologorum, 119 (2017), 3; 1
ı influences chemical composition of milk and udder health in dairy cows // Veterinarska stanica, 48 (2017), 4; 2
oligosaccharide and clinoptilolite on hematological, biochemical and gut histological parameters in weaned pigs ,

ductive status in dairy cows // Proceedings of XXVI International Congress of the Hungarian Assocition for Buiatri
favourably modulates intestinal and systemic immunity and some production parameters in weaned pigs // Vet

with the supplement of nature zeolite clinoptilolite // Meso : prvi hrvatski časopis o mesu, 15 (2013), 6; 496-499
ers of weaned piglets // 2. Workshop Feeed-to-food FP7 regpot-3 XIV Međunarodni simpozijum Tehnologija hran

oring its bactericidal and direct anti-inflammatory properties. International immunopharmacology, 124, 110920.

bacteria isolated from integumentary infections in animals. Photodiagnosis and photodynamic therapy, 40, 1031

M. P. R., Larangeira, D. F., Silva, L. P., de Carvalho, V. D. M. P., Canuto, G. A. B., ... & Estrela-Lima, A. (2025). Oxidative analysis, clinical-lal

vaquejada athlete horses. Arquivo Brasileiro de Medicina Veterinária e Zootecnia, 74(06), 1024-1038.

ИНАРНОЇ МЕДИЦИНИ. Подільський вісник: сільське господарство, техніка, економіка, (38), 203-209.

Efficacy against Postweaning Diarrheal Disease of Pigs Due to F4+ and F18+ ETEC Strains. Vet. Arhiv 2022, 92, 2

future. International journal of veterinary science and medicine, 2018, 6(1), 1–7. https://doi.org/10.1016/j.ijvsm

Farm Animal Veterinary Medicine, Japan Veterinary Medical Association: Sapporo, Japan, 2018; pp. 373–373.

ıtive health solutions. Computational and Structural Biotechnology Journal 2025, 29, 85-94. https://doi.org/10.1(

ıancy and lactation // Polish journal of veterinary sciences, 23 (2020), 1; 69-75. doi: 10.24425/pjvs.2020.132750

'/ Microporous and mesoporous materials, 292 (2020), 109766, 4. doi: 10.1016/j.micromeso.2019.109766
/ and early lactation // Research in veterinary science, 127 (2019), 57-64. doi: 10.1016/j.rvsc.2019.10.010

:y and early lactation // Proceedings of the 22th Annual Conference of the European Society for Domestic Anima

the 30th World Buiatric Congress, Sapporo, Japan. Sapporo: Japanese Society of Farm Animal Veterinary Medicir

the Hungarian Association for Buiatrics. Budimpešta: Hungarian Association for Buiatrics, 2017. str. 232-233

boratory parameters, and quality of life in treatment associated with ozone therapy in female dogs with mammary tumors. *Research in Veterir*

ıe, Japan Veterinary Medical Association, 2018. str. 373-373

Identificati	Records id	1124
Duplicates	Remaining	842
Screening	Records sc	842
Excluded	Records ex	628
Eligibility	Full-text as	214
Eligibility	Full-text ex	118
Included	Studies inc	96
