# Supplementary material for: Evidence and Clinical Applications of Natural Products in Veterinary Medicine: A Systematic Review of Clinoptilolite, Ozone Therapy, Propolis, and Phytotherapy
Source: Vet Sci. 2026 May 16;13(5):483. doi: 10.3390/vetsci13050483 (PMC13211563; doi:10.3390/vetsci13050483)
Supplement: Supplementary file 1 [file vetsci-13-00483-s001.zip › Table S2. Database_Specific Search Strategies.pdf]

Table S2. Database-Specific Search Strategies

PubMed (MeSH + Keywords):
(("Ozone Therapy"[Mesh] OR "ozone therapy"[tiab] OR ozone[tiab])
AND ("Zeolites"[Mesh] OR clinoptilolite[tiab] OR zeolite*[tiab])
AND ("Propolis"[Mesh] OR propolis[tiab] OR "bee product*"[tiab])
AND ("Phytotherapy"[Mesh] OR phytotherapy[tiab] OR phytogenic*[tiab] OR "plant extract*"[tiab])
AND ("Animals"[Mesh] OR animal*[tiab] OR livestock[tiab] OR veterinary[tiab])
AND (clinical[tiab] OR trial[tiab] OR treatment[tiab]))

Scopus:
TITLE-ABS-KEY(("ozone therapy" OR ozone) AND (clinoptilolite OR zeolite*) AND (propolis OR "bee-derived products") AND (phytotherapy OR phytogenic* OR "plant extract*") AND (veterinary OR animal* OR livestock) AND (clinical OR trial OR treatment))

Web of Science Core Collection:
TS=(("ozone therapy" OR ozone) AND (clinoptilolite OR zeolite*) AND (propolis OR "bee-derived products") AND (phytotherapy OR phytogenic* OR "plant extract*") AND (veterinary OR animal* OR livestock) AND (clinical OR trial OR treatment))

MDPI:
("ozone therapy" OR ozone) AND (clinoptilolite OR zeolite*) AND (propolis OR "bee-derived products") AND (phytotherapy OR phytogenic* OR "plant extract*") AND (veterinary OR animal* OR livestock) AND (clinical OR trial OR treatment)

Additional sources:
CAB Abstracts, Google Scholar (first 200 results screened), and manual reference screening.
