# Supplementary material for: Evidence and Clinical Applications of Natural Products in Veterinary Medicine: A Systematic Review of Clinoptilolite, Ozone Therapy, Propolis, and Phytotherapy
Source: Vet Sci. 2026 May 16;13(5):483. doi: 10.3390/vetsci13050483 (PMC13211563; doi:10.3390/vetsci13050483)
Supplement: Supplementary file 1 [file vetsci-13-00483-s001.zip › Table S3. Risk of Bias.pdf]

Supplementary Table S3. Risk of Bias Assessment of Included Studies (n = 96)

Legend:

L = Low risk | M = Moderate risk | H = High risk

Study	Selection	Performance	Detection	Attrition	Reporting	Other
1	M	H	M	L	M	M
2	M	M	M	L	M	M
3	M	M	M	L	M	M
4	L	H	M	L	L	M
5	L	H	M	L	L	M
6	M	H	M	L	M	M
7	M	M	M	L	M	M
8	L	M	L	L	L	L
9	L	M	L	L	L	L
10	L	M	L	L	L	L
11	L	M	L	L	L	L
12	M	H	M	M	M	M
13	L	M	L	L	L	L
14	M	M	M	L	M	M
15	M	M	M	L	M	M
16	M	H	M	L	M	M
17	M	H	M	L	M	M
18	M	H	M	L	M	M
19	M	H	M	L	M	M

20	L	M	L	L	L	L
21	M	M	M	L	M	M
22	M	H	M	L	M	M
23	M	H	M	L	M	M
24	M	H	M	L	M	M
25	M	M	M	L	M	M
26	M	M	M	L	M	M
27	L	M	L	L	L	L
28	L	M	L	L	L	L
29	M	M	M	L	M	M
30	M	H	M	L	M	M
31	M	H	M	L	M	M
32	M	M	M	L	M	M
33	M	H	M	L	M	M
34	M	H	M	L	M	M
35	H	H	M	M	M	M
36	M	M	M	L	M	M
37	M	H	M	L	M	M
38	M	H	M	L	M	M
39	M	H	M	L	M	M
40	M	H	M	L	M	M
41	M	H	M	L	M	M
42	H	H	M	M	M	M
43	M	H	M	L	M	M
44	L	M	L	L	L	L
45	M	M	M	L	M	M

46	H	H	M	M	M	M	
47	M	M	M	L	M	M	
48	M	M	M	L	M	M	
49	M	H	M	L	M	M	
50	M	H	M	L	M	M	
51	M	M	M	L	M	M	
52	M	M	M	L	M	M	
53	M	M	M	L	M	M	
54	M	M	M	L	M	M	
55	M	M	M	L	M	M	
56	M	M	M	L	M	M	
57	M	M	M	L	M	M	
58	M	M	M	L	M	M	
59	H	H	M	M	M	M	
60	M	M	M	L	M	M	
61	M	M	M	L	M	M	
62	M	M	M	L	M	M	
63	M	M	M	L	M	M	
64	M	M	M	L	M	M	
65	M	M	M	L	M	M	
66	M	M	M	L	M	M	
67	M	M	M	L	M	M	
68	M	M	M	L	M	M	
69	M	M	M	L	M	M	
70	M	M	M	L	M	M	
71	M	M	M	L	M	M	

72	M	M	M	L	M	M
73	M	M	M	L	M	M
74	M	M	M	L	M	M
75	M	M	M	L	M	M
76	M	H	M	L	M	M
77	M	M	M	L	M	M
78	M	H	M	L	M	M
79	M	H	M	L	M	M
80	M	H	M	L	M	M
81	M	H	M	L	M	M
82	M	M	M	L	M	M
83	M	H	M	L	M	M
84	M	H	M	L	M	M
85	M	M	M	L	M	M
86	M	H	M	L	M	M
87	M	M	M	L	M	M
88	M	M	M	L	M	M
89	M	H	M	L	M	M
90	M	H	M	L	M	M
91	M	H	M	L	M	M
92	M	H	M	L	M	M
93	M	H	M	L	M	M
94	M	M	M	L	M	M
95	M	M	M	L	M	M
96	M	M	M	L	M	M
